# Hsp90β interacts with MDM2 to suppress p53‐dependent senescence during skeletal muscle regeneration

**DOI:** 10.1111/acel.13003

**Published:** 2019-07-17

**Authors:** Min Yi He, Shui Bo Xu, Zi Hao Qu, Yue Mei Guo, Xiao Ceng Liu, Xiao Xia Cong, Jian Feng Wang, Boon Chuan Low, Li Li, Qiang Wu, Peng Lin, Shi Gui Yan, Zhang Bao, Yi Ting Zhou, Li Ling Zheng

**Affiliations:** ^1^ Department of Biochemistry and Molecular Biology, Department of Orthopaedic Surgery of the Second Affiliated Hospital Zhejiang University School of Medicine Hangzhou China; ^2^ Key Laboratory of Tissue Engineering and Regenerative Medicine of Zhejiang Province, Dr. Li Dak Sum & Yip Yio Chin Center for Stem Cell and Regenerative Medicine Zhejiang University School of Medicine Hangzhou China; ^3^ Department of Respiratory Medicine, The First Affiliated Hospital Zhejiang University School of Medicine Hangzhou China; ^4^ Mechanobiology Institute, Department of Biological Sciences National University of Singapore Singapore; ^5^ Institute of Aging Research Hangzhou Normal University Hangzhou China; ^6^ The State Key Laboratory of Quality Research in Chinese Medicine Macau University of Science and Technology Macau China; ^7^ ZJU‐UoE Institute Zhejiang University School of Medicine Hangzhou China

**Keywords:** cellular senescence, heat‐shock protein, Hsp90β, MDM2, muscle regeneration, p53, senescence, skeletal muscle

## Abstract

Cellular senescence plays both beneficial and detrimental roles in embryonic development and tissue regeneration, while the underlying mechanism remains elusive. Recent studies disclosed the emerging roles of heat‐shock proteins in regulating muscle regeneration and homeostasis. Here, we found that Hsp90β, but not Hsp90α isoform, was significantly upregulated during muscle regeneration. RNA‐seq analysis disclosed a transcriptional elevation of p21 in Hsp90β‐depleted myoblasts, which is due to the upregulation of p53. Moreover, knockdown of Hsp90β in myoblasts resulted in p53‐dependent cellular senescence. In contrast to the notion that Hsp90 interacts with and protects mutant p53 in cancer, Hsp90β preferentially bound to wild‐type p53 and modulated its degradation via a proteasome‐dependent manner. Moreover, Hsp90β interacted with MDM2, the chief E3 ligase of p53, to regulate the stability of p53. In line with these in vitro studies, the expression level of p53‐p21 axis was negatively correlated with Hsp90β in aged mice muscle. Consistently, administration of 17‐AAG, a Hsp90 inhibitor under clinical trial, impaired muscle regeneration by enhancing injury‐induced senescence in vivo. Taken together, our finding revealed a previously unappreciated role of Hsp90β in regulating p53 stability to suppress senescence both in vitro and in vivo.

## 1 INTRODUCTION

Cellular senescence is featured by cell cycle arrest that is due to the enhanced expression of two master regulatory axis, p53‐p21 and p16INK4a‐pRB (Pawlikowski, Adams, & Nelson, 2013). Since both p53 and pRB are potent tumor suppressors, senescence is recognized as a tumor‐suppressor mechanism (Campisi, Andersen, Kapahi, & Melov, 2011). Recent studies demonstrated the contribution of senescent cells in nonpathologic scenarios such as embryonic development and adult tissue regeneration (Storer et al., 2013). Moreover, both beneficial and detrimental functions of senescence have been identified in muscle repair, while the underlying mechanism remains poorly understood (Chiche et al., 2017; Demaria et al., 2014; Le Roux, Konge, Le Cam, Flamant, & Tajbakhsh, 2015). Thus, it is of interest to delineate the divergent molecular mechanisms by which senescence elicits different effects on regeneration.

In vivo activation of p53 not only led to cancer‐free mice but also resulted in premature aging and shortened lifespan (Maier et al., 2004; Tyner et al., 2002). A p53‐dependent persistent senescence in myoblasts was found to impair muscle regeneration (Le Roux et al., 2015). Moreover, the temporal regulation of p53 expression is critical for limb regeneration (Yun, Gates, & Brockes, 2013). However, the protein machinery controlling p53‐dependent senescence during tissue regeneration remains elusive. It is well documented that more than half of human cancers contain inactive p53 mutant proteins (Bieging, Mello, & Attardi, 2014; Brosh & Rotter, 2009; Trepel, Mollapour, Giaccone, & Neckers, 2010) and these mutant p53 in cancers were protected by the 90 kDa heat‐shock protein (Hsp90) (Brosh & Rotter, 2009; Taipale, Jarosz, & Lindquist, 2010). Considerable progress has thus been made to delineate the pathological function and underlying mechanism of Hsp90 in cancer. However, the physiological role of Hsp90 proteins in modulating p53‐dependent senescence during tissue homeostasis and regeneration remains elusive.

Recent work by us and others disclosed divergent roles of heat‐shock proteins in modulating myogenesis. For instance, Hsp70 interacted with MAPKAPK2/MK2 to stabilize p38MAPK and promote muscle regeneration (Fan et al., 2018). In comparison, promyogenic heat‐shock protein Hsp10/Cpn10 interacts with NPAT to promote cell proliferation (Kayani et al., 2010; Ling Zheng et al., 2015). In this study, we found an elevation of Hsp90β during muscle regeneration. Interestingly, in contrast to the notion that Hsp90 stabilizes mutant p53 in cancer (Trepel et al., 2010), knockdown of Hsp90β in myoblast led to the upregulation of wild‐type p53 and cellular senescence. Hsp90β preferentially interacted with wild‐type p53 and its master E3 ligase MDM2. Moreover, administration of Hsp90 inhibitors 17‐AAG, which is under clinical trial, and CCT018159 impaired muscle regeneration by sustaining injury‐induced senescence. Taken together, our finding highlights a previously unappreciated role of Hsp90β in regulating p53‐dependent senescence during muscle regeneration.

## 2 RESULTS

### 2.1 Depletion of Hsp90β elevates p53‐p21 axis in myoblasts

**Figure 1:**
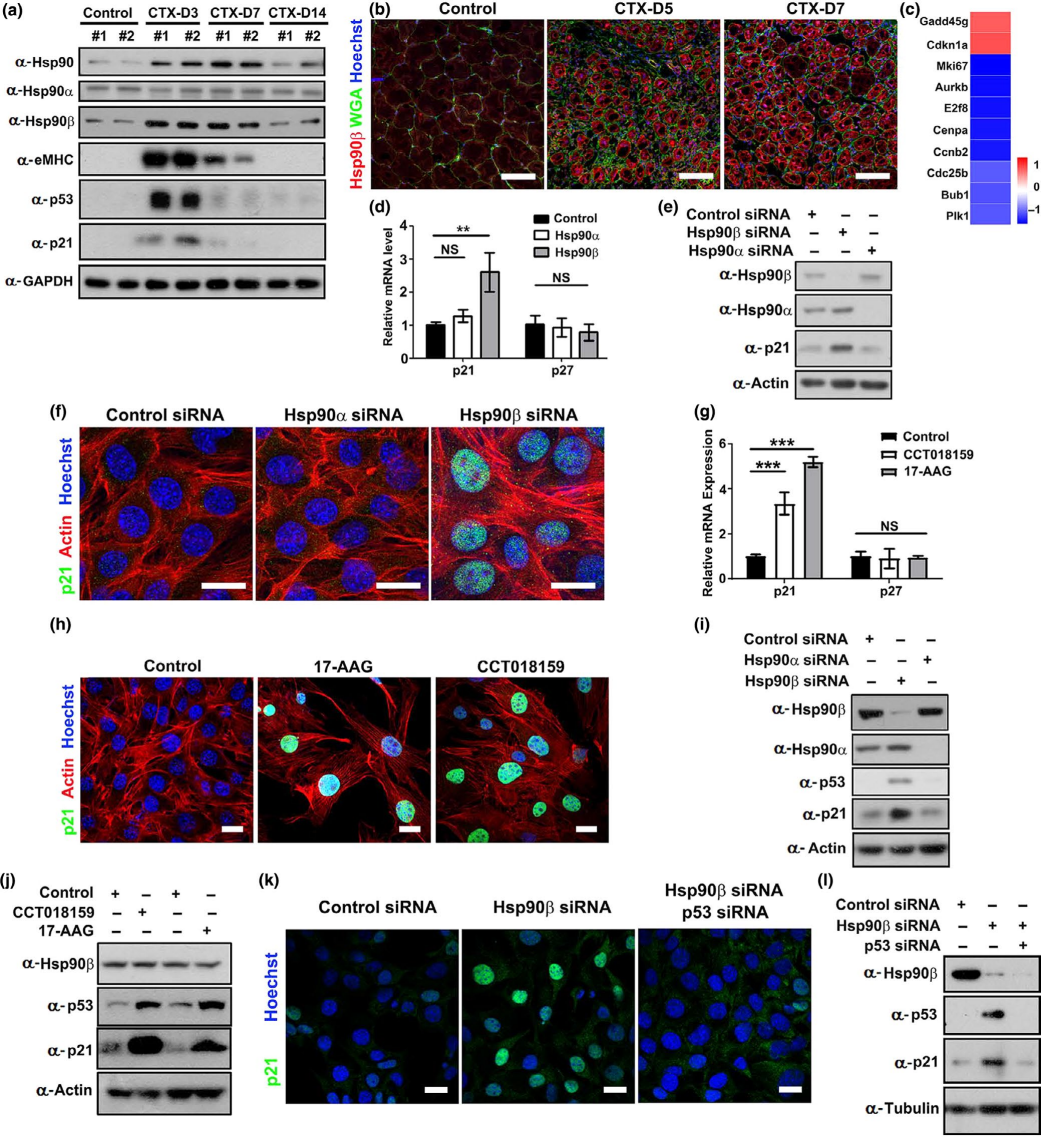
Inhibition or depletion of Hsp90β elevated p53‐p21 axis in myoblasts. (a) Tibialis anterior (TA) muscles of male mice were injected with CTX and were harvested on days 3, 7, and 14 postinjury for Western blot analysis with the indicated antibodies. *n* = 6 male mice for each group. Western blots of all samples were displayed and quantified as shown in Supporting Information Figure S1a‐c. (b) Cryosections of regenerating TA muscles on day 5 and day 7 post‐CTX (CTX‐D5 and CTX‐D7) injury were stained with Hoechst for nuclei, wheat germ agglutinin (WGA) for myofiber membrane boundaries, and Hsp90β antibody. Bar: 50μm. Enlarged images are displayed in Supporting Information Figure S1e. (c) Heatmap of RNA‐seq analysis of the genes in control or Hsp90β siRNA‐transfected C2C12 myoblasts. Blue indicates downregulated genes, while red indicates upregulated genes in Hsp90β‐depleted myoblasts. Logarithm base 2 (fold change) values were calculated from fragments per kilobase of transcript per million (FPKM) values of RNA‐seq. (d) C2C12 myoblasts transfected with control, Hsp90α, or Hsp90β siRNA for 48 hr were subjected to qPCR analysis for p21 and p27 expression (**: *p* < 0.01). (e) Control, Hsp90α, or Hsp90β siRNA‐transfected myoblasts were subjected to Western blot for p21 expression. (f) Control, Hsp90α, or Hsp90β siRNA‐transfected myoblasts were immunostained with p21 antibody. The actin filaments and nuclei were visualized by rhodamine‐conjugated phalloidin or Hoechst staining. Bar: 20μm. (g) C2C12 myoblasts treated with 17‐AAG (10nM) or CCT018159 (5nM) were subjected to qPCR analysis for p21 and p27 expression (***: *p* < 0.001). (h) Control myoblasts or myoblasts treated with 17‐AAG or CCT018159 were immunostained with p21 antibody. The actin filaments and nuclei were visualized by rhodamine‐conjugated phalloidin or Hoechst staining. Bar: 20μm. (i) C2C12 myoblasts transfected with control, Hsp90α, or Hsp90β siRNA for 48 hr were subjected to Western blot for p53 and p21 expression. (j) Control myoblasts or myoblasts treated with CCT018159 or 17‐AAG for 48 hr were subjected to Western blot for p53 and p21 expression. (k) C2C12 myoblasts transfected with control siRNA, Hsp90β siRNA, or co‐transfected with Hsp90β and p53 siRNA sequences for 48 hr were subjected to immunofluorescence analysis for p21 expression. Bar: 20 μm. (l) C2C12 myoblasts transfected with control siRNA, Hsp90β siRNA, or co‐transfected with Hsp90β and p53 siRNA sequences for 48 hr were subjected to Western blot analysis for p53 and p21 expression

To address the function of Hsp90 in muscle regeneration, we examined the temporal expression patterns of its two isoforms (Hsp90α and Hsp90β) in a widely used snake venom cardiotoxin (CTX)‐induced injury model (Charge & Rudnicki, 2004). Tibialis anterior (TA) muscles of eight‐week‐old mice were subjected to CTX injection and then allowed to recover for 3 to 14 days. After intramuscular injection of CTX, myoblasts start to proliferate and differentiate for muscle regeneration. New myofibers are normally formed 5 to 7 days afterward, and muscle construction is largely reestablished within 14 days (Charge & Rudnicki, 2004). Total Hsp90 and Hsp90β isoforms, but not Hsp90α isoform, were robustly elevated on day 3 and day 7 postinjury in which muscle regeneration occurs as evidenced by the expression of embryonic myosin heavy chain (eMHC), which is specifically upregulated during embryonic myogenesis and muscle regeneration (Figure 1a; Figure S1a‐c). The expression levels of both eMHC and Hsp90β were downregulated when active myogenesis ceased at day 14. Immunostaining verified that Hsp90β isoform, but not Hsp90α isoform, was upregulated during muscle regeneration (Figure 1b; Figure S1d‐f).

Expanded myoblasts fuse to form new myofibers for repairing during muscle regeneration (Bentzinger, Wang, & Rudnicki, 2012). We therefore examined the gene expression profile in Hsp90β‐depleted C2C12 myoblasts. RNA‐seq analysis revealed that depletion of Hsp90β in myoblast resulted in a upregulation of p21 CiP1/Waf1, a universal Cdk/cyclin inhibitor (Bertoli, Skotheim, & de Bruin, 2013), and a p21 interacting protein Gadd45, which also induces cell cycle arrest (Carrier et al., 1999) (Figure 1c). The elevation of p21 on both mRNA and protein levels was verified by RT–qPCR analysis and Western blot (Figure 1d‐e). Immunofluorescence analysis revealed the upregulation of p21 in nuclei by Hsp90β depletion (Figure 1f). We further treated myoblasts with Hsp90 inhibitor 17‐AAG, a Hsp90 inhibitor which entered clinical trials (Trepel et al., 2010), or a Hsp90β‐specific inhibitor CCT018159 (Sharp et al., 2007). Both inhibitors upregulated the expression of p21 in myoblasts (Figure 1g‐h).

Since p53 acts as a major regulator for p21 transcription (Bieging et al., 2014), we hypothesized that downregulation of Hsp90β in myoblast might enhance p53 expression. Although it is well documented that downregulation of Hsp90 leads to degradation of mutant p53 in cancer (Brosh & Rotter, 2009; Taipale et al., 2010), Western blot analysis demonstrated that depletion of Hsp90β, but not Hsp90α, significantly enhanced the expression of p53 in myoblasts (Figure 1i). Likewise, treatment of 17‐AAG or CTT018159 also upregulated the expression of p53 in myoblast (Figure 1j). Moreover, knockdown of p53 inhibited p21 upregulation in either Hsp90β‐depleted or Hsp90 inhibitor‐treated myoblasts (Figure 1k‐l; Figure S1g‐h). Thus, we concluded that Hsp90β regulates p53‐p21 axis in myoblast.

### 2.2 Hsp90β suppresses p53‐dependent cellular senescence in myoblasts

**Figure 2:**
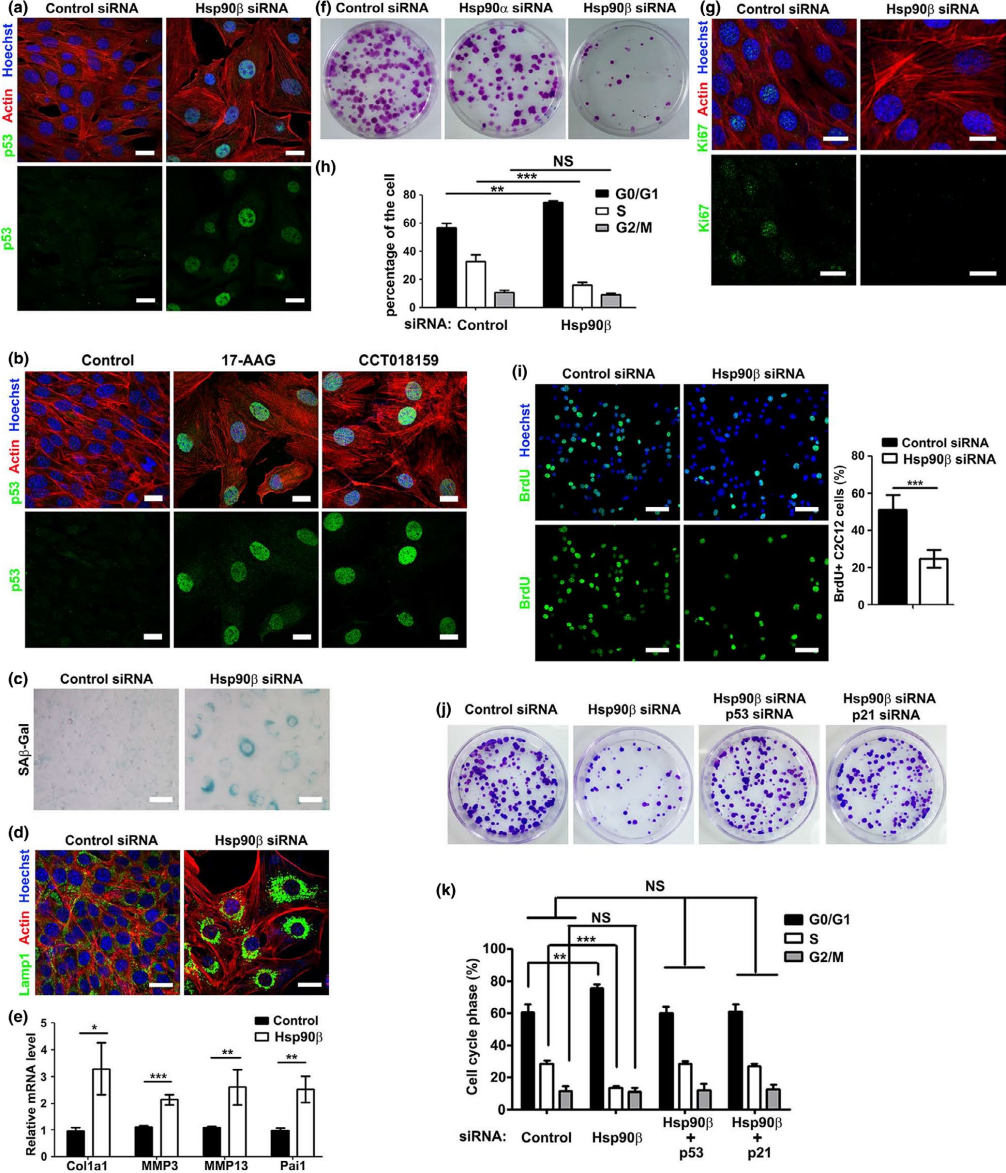
Depletion of Hsp90β led to p53‐dependent cellular senescence in myoblasts. (a) C2C12 myoblasts were continuously transfected with control or Hsp90β siRNA for three passages followed by immunostaining with p53 antibody. The actin filaments and nuclei were visualized by rhodamine‐conjugated phalloidin or Hoechst staining. Bar: 20μm. Cell size was quantified as shown in Figure S2a. (b) C2C12 myoblasts treated with DMSO, 17‐AAG, or CCT018159 for two days were immunostained with p53 antibody. Bar: 20μm. Cell size was quantified as shown in Figure S2b. (c) C2C12 myoblasts continuously transfected with control or Hsp90β siRNA for five passages were subjected to SA‐βGal staining. Bar: 50μm. (d) Control or Hsp90β siRNA‐transfected myoblasts for three passages were immunostained with Lamp1 antibody. Bar: 20μm. (e) Control or Hsp90β siRNA‐transfected myoblasts for three passages were subjected to qPCR for analyzing Collagen I, MMP3, MMP13, and Pai1 expression. (f) Control, Hsp90α or Hsp90β siRNA‐transfected myoblasts were subjected to colony formation assay. (g) Control or Hsp90β siRNA‐transfected myoblasts were stained with Ki67 antibody to analyze proliferation ability. Bar: 20μm. (h) Control or Hsp90β siRNA‐transfected myoblasts were subjected to FACS analysis to determine the percentage of cells at different cell phases. (i) Control or Hsp90β siRNA‐transfected myoblasts were subjected to BrdU incorporation analysis to measure cell proliferation ability. Bar: 50μm. (j) Control siRNA, Hsp90β siRNA, p53 siRNA, and p21 siRNA sequences were transfected into C2C12 myoblasts in the indicated combination and were subjected to colony formation assay to determine the cell proliferation ability. (k) Myoblasts transfected with siRNA sequences in the indicated combination were subjected to FACS analysis to determine the percentage of cells at different cell phases. All the experiments shown are representative of at least three biological replicates. Statistical analysis was performed with Student's *t* test (*p < 0.05, **p < 0.01, ***p < 0.001)

Since p53‐p21 axis represents a pivotal pathway for cellular senescence (Pawlikowski et al., 2013), we hypothesized that Hsp90β suppresses cellular senescence in myoblasts. This hypothesis was supported by the observation that either siRNA‐mediated depletion or inhibition of Hsp90β led to enlarged cell size, which is a hallmark of cellular senescence (Munoz‐Espin & Serrano, 2014) (Figure 2a‐b; Figure S2a‐b). In line with this, knockdown or inhibition of Hsp90β in myoblasts significantly increased senescence‐associated β‐galactosidase (SA‐βGal)‐positive myoblasts (Figure 2c; Figure S2c) and enhanced Lamp1 staining (Figure 2d; Figure S2d), two biomarkers for cellular senescence (Munoz‐Espin & Serrano, 2014). Moreover, senescence‐related genes including Collagen I, MMP3, MMP13, and Pai1 were also upregulated in Hsp90β‐depleted or Hsp90 inhibitor‐treated myoblasts (Figure 2e; Figure S2e). Since senescent cells are characterized by irreversible cell cycle arrest (Munoz‐Espin & Serrano, 2014), we also checked cell cycle and proliferation in myoblasts. Reduced colony number, decreased Ki67 staining, and G1/S phase arrest were observed in Hsp90β‐depleted or Hsp90 inhibitor‐treated myoblasts (Figure 2f‐h; Figure S2f‐h). Furthermore, knockdown of Hsp90β reduced BrdU staining (Figure 2i). To verify whether the defective cell proliferation in Hsp90β‐depleted myoblasts is indeed due to the upregulation of p53‐p21 axis, we knocked down either p53 or p21 in Hsp90β‐depleted C2C12 myoblasts. Depletion of p21 or p53 could rescue cell proliferation and release cell cycle arrest in Hsp90β‐depleted myoblasts (Figure 2j‐k). Taken together, these data demonstrated that Hsp90β regulates cellular senescence via the p53‐p21 axis.

### 2.3 Hsp90β regulates the stability of wild‐type p53 in myoblast

**Figure 3:**
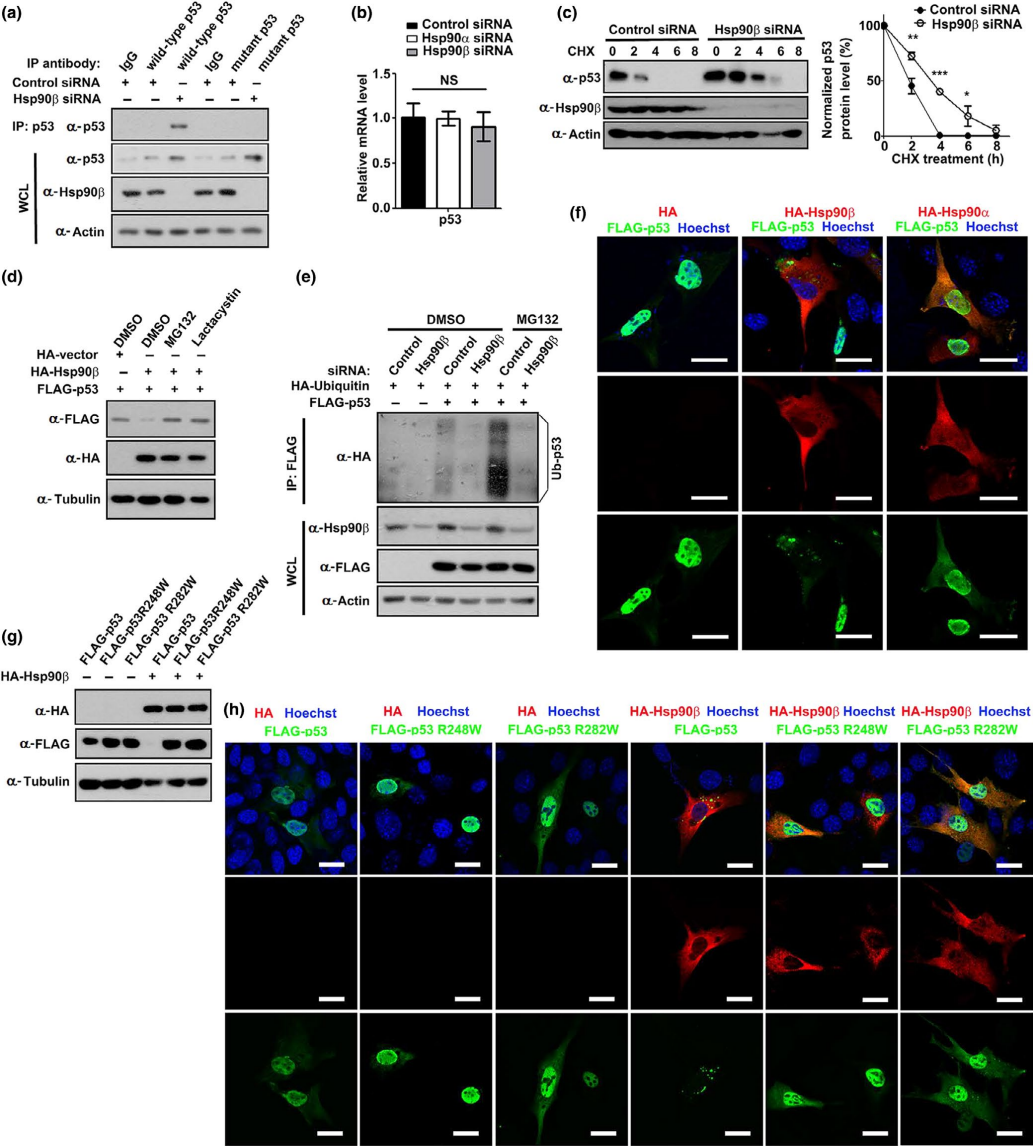
Hsp90β regulates the degradation of wild‐type p53 in myoblast. (a) Lysates of C2C12 myoblasts transfected with control or Hsp90β siRNA were immunoprecipitated with control IgG or antibodies that recognizes wild‐type or mutant p53, and then Western‐blotted with p53 antibody. (b) C2C12 myoblasts transfected with control, Hsp90α, or Hsp90β siRNA were subjected to RT–qPCR for analyzing p53 expression. (c) C2C12 cells were transfected with control or Hsp90β siRNA for 48 hr followed by treating with cycloheximide (CHX) (100 µg/ml). Cell lysates collected at the indicated times were subjected to Western blot analysis, and the expression levels of p53 were quantified. Data shown are representative of three biological replicates. Statistical analysis was performed with Student's *t* test (*p < 0.05, **p < 0.01, and ***p < 0.001). (d) C2C12 myoblasts were co‐transfected with FLAG‐p53 and HA vector or HA‐Hsp90β for 30 hr followed by incubating with DMSO (control), the protease inhibitor MG132 (5 µM), or lactacystin (10 µM) for 6 hr. Cell lysates were subjected to Western blot analysis with the indicated antibodies. (e) C2C12 cells were transfected with control or Hsp90β siRNA for 12 hr followed by transfecting with HA‐ubiquitin and FLAG‐p53 in the indicated combination for 30 hr. After 6‐hr treatment of MG132, p53 ubiquitination was determined by immunoprecipitation with an anti‐FLAG antibody and immunoblotted with HA antibody. (f) C2C12 myoblasts were co‐transfected with FLAG‐p53 and HA vector, HA‐Hsp90α, or HA‐Hsp90β for 36 hr followed by immunofluorescence analysis. Nuclei were visualized by Hoechst staining. Bar: 20μm. (g) FLAG‐p53, FLAG‐p53 R248W, and FLAG‐p53 R282W were co‐transfected with HA vector or HA‐Hsp90β into C2C12 myoblasts in the indicated combination for 36 hr followed by Western blot analysis. (h) FLAG‐p53, FLAG‐p53 R248W, and FLAG‐p53 R282W were co‐transfected with HA vector or HA‐Hsp90β into C2C12 myoblasts in the indicated combination for 36 hr followed by immunofluorescence analysis. Nuclei were visualized by Hoechst staining. Bar: 20μm

Our finding that depletion of Hsp90β elevated p53 expression is surprising since previous studies demonstrated that inhibition of Hsp90 resulted in degradation of p53 mutant proteins in cancer (Trepel et al., 2010). Thus, it is of interest to verify the elevated p53 is wild‐type or mutant protein. The folding structure of mutant p53 is different from that of wild‐type p53 (Bieging et al., 2014). Therefore, two antibodies specifically target wild‐type or mutant p53 were used for immunoprecipitation analysis (Gannon, Greaves, Iggo, & Lane, 1990). As shown in Figure 3a, only wild‐type p53 was identified in the precipitate of Hsp90β‐depleted myoblasts, demonstrating that Hsp90β specifically regulates the expression of wild‐type p53. Likewise, inhibition of Hsp90 only elevated the expression of wild‐type p53 (Figure S3a). Moreover, the transcriptional expression levels of p53 remained unchanged in Hsp90β‐depleted myoblasts (Figure 3b), indicating that Hsp90β regulates p53 stability. We therefore tested the half‐life of p53 in Hsp90β‐depleted or 17‐AAG‐treated myoblasts. New protein synthesis was prevented by treatment with the translational inhibitor cycloheximide in C2C12 myoblasts. Knockdown of Hsp90β or 17‐AAG treatment delayed the degradation of p53 (Figure 3c; Figure S3b). In line with this, forced expression of Hsp90β reduced ectopically expressed p53 levels (Figure 3d). The proteasome inhibitor MG132 or lactacystin treatment could restore p53 levels in Hsp90β‐expressing myoblasts (Figure 3d), which confirmed that Hsp90β regulated the proteasome‐dependent degradation of p53. Poly‐ubiquitination of p53 is required for its proteasome‐mediated degradation (Nagata et al., 1999). p53 ubiquitination levels in control or Hsp90β siRNA‐transfected myoblasts were examined by co‐transfection with HA–ubiquitin. As shown in Figure 3e, depletion of Hsp90β significantly reduced p53 ubiquitination.

Since the nucleocytoplasmic shuttling of p53 is essential for regulating its degradation (O'Keefe, Li, & Zhang, 2003), we examined whether Hsp90β modulates the cellular localization of p53. Ectopically expressed p53 mainly localized in nuclei, while overexpression of Hsp90β, but not Hsp90α, reduced p53 expression and led to the translocation of p53 to cytosol (Figure 3f; Figure S3c). Given the finding that depletion of Hsp90β only elevates wild‐type p53 (Figure 3a), we examined whether Hsp90β specifically promoted the degradation of wild‐type p53, but not mutant p53. Two tumor‐derived p53 mutants R248W and R282W, which are also known as “hotspot mutant” (Brosh & Rotter, 2009), failed to be suppressed by Hsp90β (Figure 3g,h). Furthermore, both R248W and R282W mutants retained in nuclei in the presence of Hsp90β (Figure 3h). All these findings favor a notion that Hsp90β regulates the degradation of wild‐type p53 in myoblast.

### 2.4 Hsp90β modulates p53 stability through interacting with MDM2

**Figure 4:**
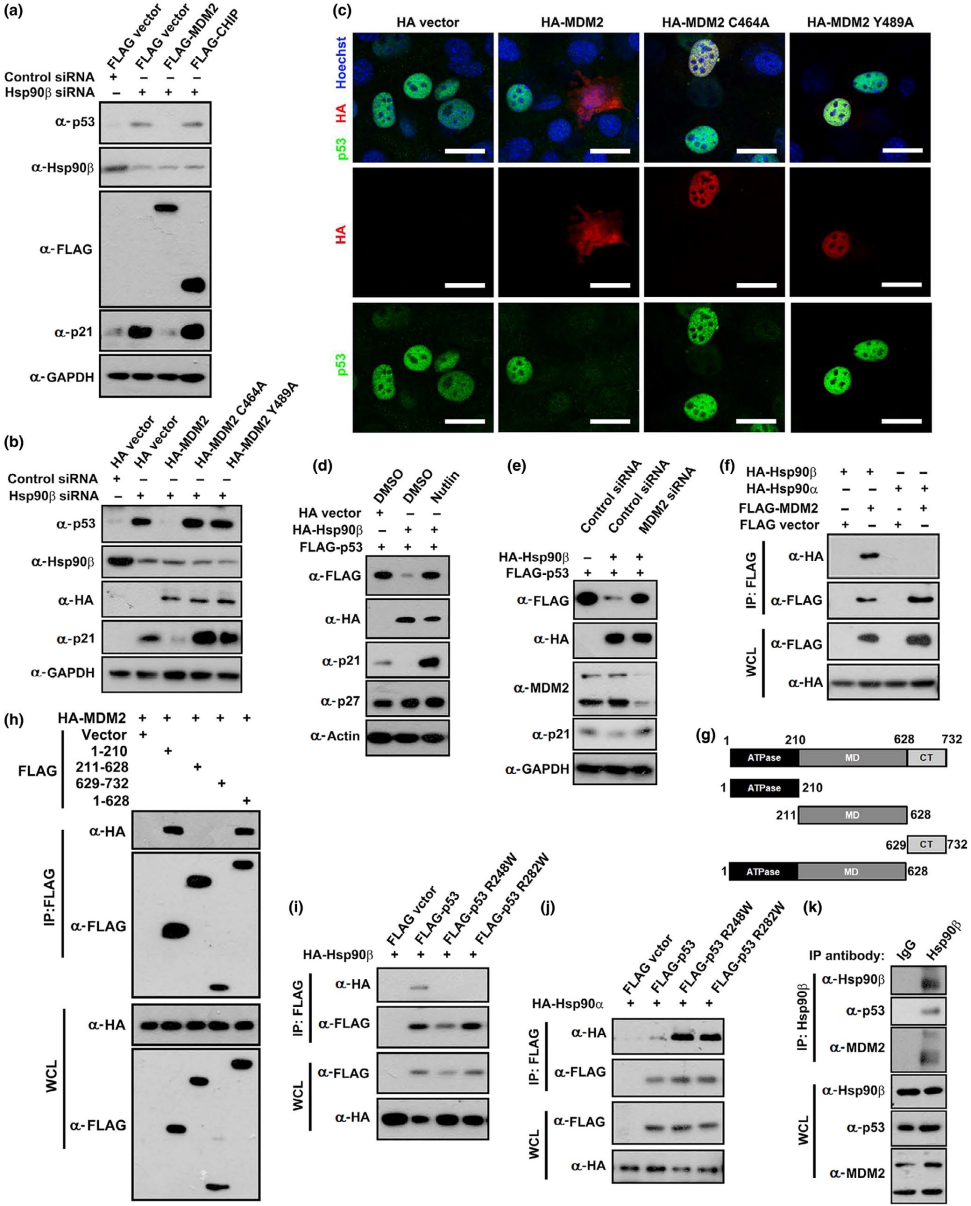
Hsp90β interacts with MDM2 to modulate wild‐type p53 stability. (a) C2C12 cells were transfected with control or Hsp90β siRNA for 12 hr followed by transfecting with FLAG vector, FLAG‐MDM2, or FLAG‐CHIP in the indicated combination. The expression levels of p53 and Hsp90β were determined by Western blot analysis. (b) C2C12 cells were transfected with control or Hsp90β siRNA for 12 hr followed by transfecting with HA vector, HA‐MDM2, HA‐MDM2 C464A, or HA‐MDM2 Y489A in the indicated combination. The expression levels of p53 and Hsp90β were determined by Western blot analysis. (c) C2C12 cells were transfected with Hsp90β siRNA for 12 hr followed by transfecting with HA vector, HA‐MDM2, HA‐MDM2 C464A, or HA‐MDM2 Y489A in the indicated combination. Cells were analyzed by immunofluorescence staining. Nuclei were visualized by Hoechst staining. Bar: 20μm. (d) C2C12 myoblasts were co‐transfected with FLAG‐p53 and HA vector or HA‐Hsp90β for 24 hr followed by incubating with DMSO (control) or the MDM2 inhibitor Nutlin (10 µM) for 12 hr. Cell lysates were subjected to Western blot analysis with the indicated antibodies. (e) C2C12 myoblasts were transfected with control or MDM2 siRNA for 12 hr followed by transfecting with FLAG‐p53 and HA vector or HA‐Hsp90β for 36 hr followed by Western blot analysis with the indicated antibodies. (f) FLAG vector, FLAG‐MDM2, HA‐Hsp90α, and HA‐Hsp90β were co‐transfected into C2C12 myoblasts in the indicated combination for 24 hr followed by immunoprecipitation analysis. (g) Schematic diagram of Hsp90β and its mutants. (h) Lysates of 293T cells transiently transfected with HA‐tagged MDM2 and FLAG‐tagged Hsp90β or its various mutant expression vectors as shown in (g) were immunoprecipitated (IP) with anti‐FLAG beads and then Western‐blotted with FLAG or HA antibodies. (i) FLAG vector, FLAG‐p53, FLAG‐p53 R248W, and FLAG‐p53 R282W were co‐transfected with HA‐Hsp90β into C2C12 myoblasts in the indicated combination followed by immunoprecipitation analysis. (j) FLAG vector, FLAG‐p53, FLAG‐p53 R248W, and FLAG‐p53 R282W were co‐transfected with HA‐Hsp90α into C2C12 myoblasts in the indicated combination followed by immunoprecipitation analysis. (k) Lysates of C2C12 myoblasts were immunoprecipitated with control IgG or Hsp90β‐specific antibody, and then Western‐blotted with the indicated antibodies

E3 ubiquitin ligase MDM2 is a chief negative regulator of p53 (Wade, Wang, & Wahl, 2010). Moreover, the chaperone‐associated ubiquitin ligase CHIP can also target p53 for proteasomal degradation (Esser, Scheffner, & Hohfeld, 2005). We thus tested whether Hsp90β regulates p53‐p21 axis via MDM2 or CHIP. Ectopic expression of MDM2, but not CHIP, significantly suppressed the upregulation of p53 and p21 in Hsp90β‐depleted myoblasts (Figure 4a). In contrast, MDM2 C464A and MDM2 Y489A mutants, which loss the E3 ligase function (Wade et al., 2010), failed to suppress p53 and p21 expression (Figure 4b). Immunofluorescence staining verified that overexpression of HA‐MDM2, but not MDM2 C464A or MDM2 Y489A mutant, suppressed the elevation of p53 in Hsp90β‐depleted myoblasts (Figure 4c). These data indicated that MDM2 is required for Hsp90β‐dependent regulation of p53 stability. We further verified this postulation by inhibiting or depleting of MDM2 in Hsp90β‐overexpressed myoblasts. Hsp90β could not suppress p53 expression in either MDM2 inhibitor Nutlin‐treated myoblasts or MDM2‐depleted myoblasts (Figure 4d‐e), indicating that Hsp90β modulates proteasome‐mediated degradation of p53 in a MDM2‐dependent manner.

We next assessed the interaction between Hsp90β and MDM2. FLAG‐tagged MDM2 only coimmunoprecipitated HA‐tagged Hsp90β, but not Hsp90α (Figure 4f). Likewise, wild‐type p53 preferentially interacted with HA‐tagged Hsp90β (Figure S4a). To identify the region within Hsp90β for binding to MDM2, a series of Hsp90β mutants were prepared (Figure 4g). The two constructs containing N‐terminus ATPase domain (amino acids 1–210 and 1–628) interacted with MDM2 (Figure 4h). Thus, we created a Hsp90β D88N mutant, in which the conserved Asp in the ATP‐binding domain was replaced by Asn (Miao et al., 2008), to examine whether the ATPase activity is required for Hsp90β to recruit MDM2 or p53. Both MDM2 and p53 interact with wild‐type Hsp90β, but not the Hsp90β D88N mutant (Figure S4b‐c). Interestingly, Hsp90β only interacted with wild‐type MDM2, but not with MDM2 C464A or MDM2 Y489A mutant (Figure S4d). We further compared the interaction profiles of Hsp90α and Hsp90β isoforms toward wild‐type or mutant p53. Figure 4i,j shows that Hsp90α favorably bound mutant p53, while Hsp90β preferentially interacted with wild‐type p53. In addition, lysates from myoblasts were immunoprecipitated with Hsp90β antibodies and endogenous Hsp90β, MDM2, and p53 were found in the precipitate (Figure 4k), suggesting the presence of Hsp90β‐MDM2‐p53 complex.

### 2.5 Hsp90β inhibits senescence in vivo

**Figure 5:**
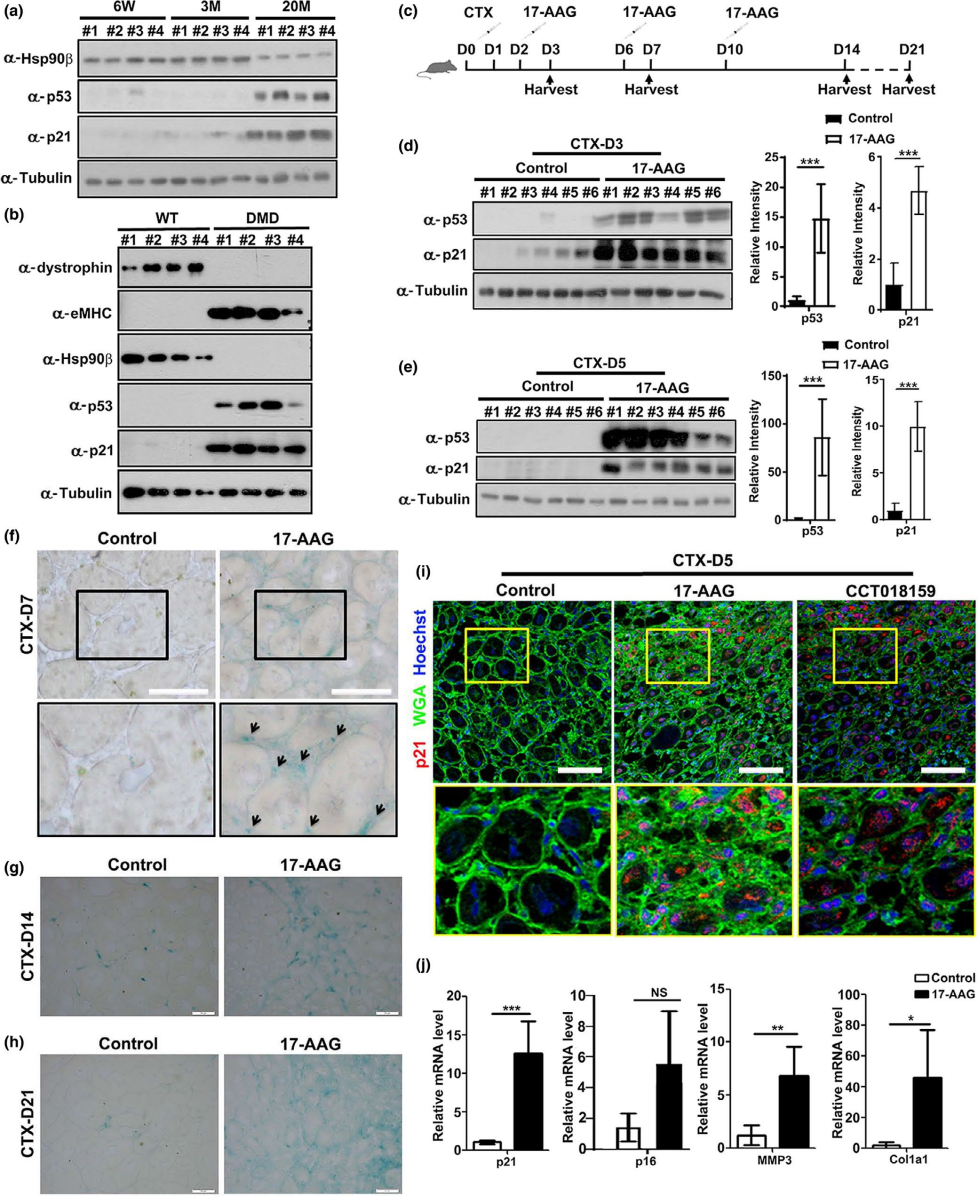
Hsp90 inhibitor 17‐AAG induced p53‐dependent senescence in vivo. (a) TA muscles from four (#1‐#4) 6‐week‐old (6W), 3‐month‐old (3M), or 20‐month‐old (20M) male mice were subjected to Western blot analysis to determine the expression levels of Hsp90β, p53, and p21. (b) TA muscles from four (#1‐#4) control or DMD male mice were subjected to Western blot analysis to determine the expression levels of Hsp90β, p53, p21, and eMHC. (c) Injection scheme for control DMSO or 17‐AAG injection into CTX‐injured mouse TA muscles. (d) TA muscles with DMSO or 17‐AAG injection from six male mice (#1‐#6) for each group were harvested on day 3 post‐CTX injury for Western blot analysis. The expression levels of p53 and p21 were quantified. (e) TA muscles with DMSO or 17‐AAG injection from six male mice (#1‐#6) for each group were harvested on day 5 post‐CTX injury for Western blot analysis. The expression levels of p53 and p21 were quantified. (f) Cryosections of regenerating TA muscles on day 7 post‐CTX injury from control or 17‐AAG‐injected male mice were subjected to SA‐βGal staining. Bar: 50μm. (g) Cryosections of regenerating TA muscles on day 14 post‐CTX injury from control or 17‐AAG‐injected male mice were subjected to SA‐βGal staining. Bar: 50μm. (h) Cryosections of regenerating TA muscles on day 21 post‐CTX injury from control or 17‐AAG‐injected male mice were subjected to SA‐βGal staining. Bar: 50μm. (i) Cryosections of regenerating TA muscles on day 5 post‐CTX injury from control, 17‐AAG‐, or CTT018159‐injected male mice were subjected to p21 staining. Bar: 50μm. (j) Cryosections of regenerating TA muscles on day 14 post‐CTX injury from control or 17‐AAG‐injected mice were subjected to RT–qPCR analysis for expression of p21, p16, MMP3, and Collagen I. Data shown are representative of three biological replicates. Statistical analysis was performed with Student's *t* test (*p < 0.05, **p < 0.01, and ***p < 0.001)

The above results support the roles of Hsp90β in inhibiting p53‐dependent cellular senescence through a MDM2‐dependent manner. We subsequently asked whether Hsp90β also modulates senescence in vivo by firstly examining its expression in young, adult, and aged mice. The expression levels of p53 and p21 were much higher in TA muscles from aged mice (20 months old) than those from young (6 weeks old) and adult (3 months old) mice (Figure 5a). Importantly, the elevation of p53‐p21 axis correlates with the downregulation of Hsp90β in aged mice muscle (Figure 5a). Furthermore, we compared skeletal muscles from wild‐type mice and dystrophic muscles from mdx (X‐linked muscular dystrophic) mice, which were featured by a pathologically active muscle degeneration and higher levels of p53 (Dogra, Srivastava, & Kumar, 2008; Nowak & Davies, 2004). Significant lower levels of Hsp90β, correlating with the elevated levels of p53‐p21 axis, were found in TA muscles from mdx mice (Figure 5b).

The above findings prompted us to further examine whether inhibition of Hsp90β could promote senescence in vivo. We injected the TA muscle of mice with CTX to analyze injury‐induced senescence (Le Roux et al., 2015). During the regenerating process, TA muscles were injected with 17‐AAG and samples were collected at 3, 5, 7, 14, and 21 days postinjection for senescence evaluation (Figure 5c). Comparing to control mice, robust upregulation of p53 and p21 was observed in muscles from 17‐AAG‐injected mice on both days 3 and 5 (Figure 5d,e). Likewise, injection of CCT018159 also led to the upregulation of both p53 and p21 in regenerating muscles (Figure S5a and S5b). We next carried out SA‐βGal staining to verify whether 17‐AAG indeed enhanced senescence in muscle. As shown in Figure 5f, SA‐βGal staining in mononucleated cells was identified in 17‐AAG‐injected regenerating muscle on day 7 post‐CTX injury. Moreover, more intensive staining of SA‐βGal was detected in 17‐AAG‐ and CCT018159‐injected muscles on day 14 and day 21 post‐CTX injury (Figure 5g‐h; Figure S5c–d). Comparing to control regenerating muscles, both 17‐AAG‐ and CCT018159‐injected muscles displayed elevated expression of endogenous p21 (Figure 5i). In addition, senescence genes including Collagen I, MMP3, and p21 were upregulated in 17‐AAG‐ or CCT018159‐injected muscles on day 14 post‐CTX injury (Figure 5j; Figure S5e). Taken together, these findings support a conclusion that administration of Hsp90 inhibitor in muscle enhanced injury‐induced senescence.

### 2.6 Hsp90β inhibitor 17‐AAG impairs muscle regeneration

**Figure 6:**
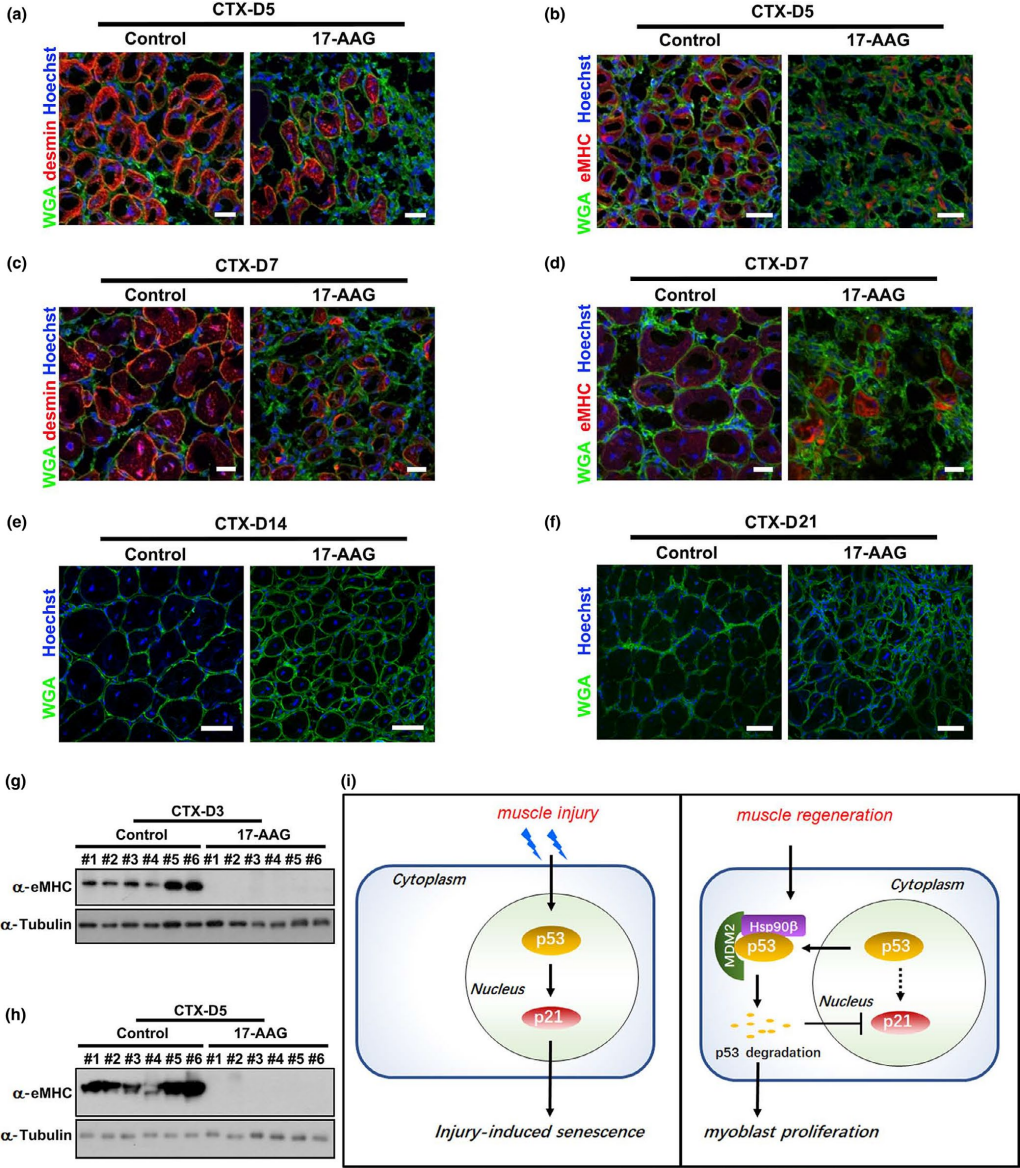
Inhibition of Hsp90 by 17‐AAG impaired muscle regeneration. (a) Cryosections of regenerating TA muscles on day 5 post‐CTX injury from control or 17‐AAG‐injected mice were stained with Hoechst for nuclei, WGA for myofiber membrane boundaries, and desmin antibody for newly formed myofibers. Bar: 50μm. (b) Cryosections of regenerating TA muscles on day 5 post‐CTX‐5 injury from control or 17‐AAG‐injected mice were stained for nuclei, myofiber membrane boundaries, and eMHC. Bar: 50μm. (c) Cryosections regenerating TA muscles on day 7 post‐CTX injury from control or 17‐AAG‐injected mice were stained for nuclei, myofiber membrane boundaries, and desmin. Bar: 50μm. (d) Cryosections of regenerating TA muscles on day 7 post‐CTX injury from control or 17‐AAG‐injected mice were stained with for nuclei, myofiber membrane boundaries, and eMHC. Bar: 50μm. (e) Cryosections of regenerating TA muscles on day 14 CTX from control or 17‐AAG‐injected mice were stained with Hoechst and WGA. Bar: 100μm. (f) Cryosections of regenerating TA muscles on day 21 post‐CTX injury from control or 17‐AAG‐injected mice were stained with Hoechst and WGA. Bar: 100μm. (g) TA muscles with DMSO or 17‐AAG injection from six male mice (#1‐#6) for each group were harvested on day 3 post‐CTX injury for Western blot analysis with eMHC. (h) TA muscles with DMSO or 17‐AAG injection from six male mice (#1‐#6) for each group were harvested on day 5 post‐CTX injury for Western blot analysis with eMHC. (i) Model depicting the regulatory mechanism for p53‐dependent senescence by Hsp90β during muscle regeneration. Injury induced the elevation of p53‐p21 axis in myoblasts and thereafter resulted in cellular senescence and impaired cell proliferation. During muscle regeneration, Hsp90β was upregulated to interact with E3 ligase MDM2 for initiating proteasome‐dependent p53 degradation. The transcription of p21 was subsequently reduced. Hence, the injury‐induced senescence was suppressed by Hsp90β to release cell cycle arrest of myoblasts and ensure the completing of muscle regeneration

Hsp90 has been recognized as a cancer therapeutic target, and more than ten Hsp90 inhibitors are undergoing clinical evaluation (Kim et al., 2009). Our above finding that Hsp90β suppressed senescence in vivo strongly suggests that application of Hsp90 inhibitors might impair muscle regeneration. To validate this potential side effect of Hsp90 inhibitor, we evaluated muscle regeneration by assessing the expression of regeneration markers in control and 17‐AAG‐treated muscles. Beside eMHC, the expression of desmin, an intermediate filament protein which is expressed in newly formed myofibers during muscle regeneration and myogenesis (Fan et al., 2018), was also examined. Immunostaining disclosed smaller cross‐sectional area (CSA) of regenerating myofibers and obviously reduced eMHC and desmin expression levels in 17‐AAG‐injected muscles on day 5 post‐CTX injection (Figure 6a,b; Figure S6a). Consistently, smaller CSA and lower expression of eMHC and desmin were also observed in 17‐AAG‐injected regenerating TA muscles on day 7 post‐CTX injection (Figure 6c,d; Figure S6a). On days 14 and 21 post‐CTX injection, the CSA of myofibers in 17‐AAG‐treated mice was smaller than that in control mice (Figure 6e‐f; Figure S6a). Injection of CCT018159 also suppressed the expression of eMHC and desmin and reduced the CSA of regenerating myofibers (Figure S6b‐S6g). Western blot analysis verified that 17‐AAG and CCT018159 inhibited eMHC expression in regenerating muscles (Figure 6g,h; Figure S6h and i). Moreover, both Sirius Red and Collagen I staining suggested enhanced fibrosis levels in 17‐AAG‐injected muscles (Figure S6j and k). In summary, our study indicates that Hsp90β is upregulated upon muscle injury to enhance p53 degradation through a MDM2‐dependent manner and thereafter inhibits p53‐dependent senescence during muscle regeneration (Figure 6i).

## 3 DISCUSSION

Skeletal muscle acts as a key regulator of systemic aging in humans (Demontis, Piccirillo, Goldberg, & Perrimon, 2013; Stearns‐Reider et al., 2017). The negative effects of senescence on skeletal muscle were recognized since loss of muscle mass during aging results in frailty and decrease in life qualify (Egerman & Glass, 2014). Reduction of quiescent muscle stem cells through senescence leads to the decline in muscle regeneration in aged mice (Sousa‐Victor et al., 2014). It is noteworthy that the senescence‐associated secretory phenotype (SASP) plays a key role in regulating the beneficial action of senescence during tissue regeneration (Mosteiro et al., 2016). Notably, transient, but not aberrant or prolonged, exposure to the SASP enhances stemness and induces cell plasticity, both of which are beneficial for regeneration (Ritschka et al., 2017). However, a p53‐dependent persistent senescence impairs muscle repair (Le Roux et al., 2015), indicating that the accurate temporal regulation of p53‐induced senescence is pivotal for ensuring accomplishment of muscle regeneration. Interestingly, a recent report showed that activated Notch‐p53 is important for the expansion of muscle stem cell in aged animal (Liu et al., 2018). Moreover, p53 also regulates the balance between myoblast differentiation and quiescence (Flamini et al., 2018). These findings indicate that the roles of p53 in modulating muscle homeostasis are complicated. Here, our studies revealed the essential roles of Hsp90β in suppressing p53‐dependent senescence in myoblasts (Figures 2, 3). It is noteworthy that inhibition of Hsp90 upregulated p53‐p21 axis, but not p16, in regenerating muscles (Figure 5j; Figure S5e). Moreover, enhanced p53‐dependent senescence was found in both mononucleated cells and myofibers during muscle regeneration (Figure 5), suggesting that our proposed model is also operative in satellite cells. These findings highlight the pivotal roles of Hsp90β in the temporal regulation of p53‐dependent senescence during regeneration. Our studies therefore support that Hsp90β serves as a potential therapeutic target for muscle injury.

Hsp90 plays critical roles in many protective cellular processes and maturation of key signaling proteins (Taipale et al., 2010). Moreover, HSP90 chaperone machinery was manipulated by cancer cells to protect mutated p53 from degradation (Trepel et al., 2010). Due to their aberrant conformations, p53 mutant proteins exhibit extended interactions with Hsp90. In line with this, our studies also demonstrated that Hsp90α preferentially bound mutant p53 (Figure 4i). In contrast, Hsp90β displayed a distinct interaction pattern that favorably immunoprecipitated wild‐type p53 (Figure 4h). Moreover, comparing to the previous finding that Hsp90 stabilizes mutant p53, our study exhibits that Hsp90β promoted the degradation of wild‐type p53 in a MDM2‐dependent manner (Figure 4a‐e). Although it is believed that Hsp90α is heat‐inducible while Hsp90β is constitutively expressed (Taipale et al., 2010), we displayed the dynamic expression of Hsp90β, but not Hsp90α, during muscle regeneration (Figure 1a,b). The two similar Hsp90 isoforms were thought to be redundant (Sreedhar, Kalmar, Csermely, & Shen, 2004), while other studies suggest functional divergence of the two proteins (Sanchez, 2012). Our finding thus provided an example displaying the different cellular functions elicited by two Hsp90 isoforms.

Given its central role in protecting oncogenic proteins, Hsp90 serves as an attractive target for cancer treatment (Trepel et al., 2010). Considerable progress has been made in identifying Hsp90 inhibitors for clinical trials. Among 13 Hsp90 inhibitors undergoing clinical evaluation, 17‐AAG is undergoing Phase III evaluation (Kim et al., 2009). Here, we demonstrated that 17‐AAG undermined muscle regeneration by inducing senescence in vivo (Figures 5 and 6), indicating a potential side effect of this inhibitor. Thus, a cautious approach is required when Hsp90 inhibitors are used in cancer treatment, particularly when the patients have muscle‐related diseases. Alternatively, discovery and validation of isoform‐specific Hsp90 inhibitor is of importance.

There are ample data showing the contribution of heat‐shock proteins in maintaining skeletal muscle homeostasis. Hsp10/Cpn10 protects skeletal muscles against contraction‐induced damage (Kayani et al., 2010). Our study indicates that this promyogenic effect is due to its interaction with NPAT/p220 to enhance cell proliferation (Ling Zheng et al., 2015). Likewise, Hsp27 attenuates disuse‐induced muscle atrophy via inhibiting NF‐kappaB signaling (Dodd, Hain, Senf, & Judge, 2009). Hsp70 also prevents skeletal muscle atrophy and protects against muscle damage (Gehrig et al., 2012), and we recently demonstrated that differentiation‐induced Hsp70 stabilizes p38MAPK via interacting with MAPKAPK2 during muscle regeneration (Fan et al., 2018). Here, we delineated that Hsp90β acts as senescence suppressor by regulating p53 turnover during muscle regeneration. This finding supports the notion that loss of proteostasis is related to aging (Kaushik & Cuervo, 2015). Moreover, it is of interest to delineate the upstream signaling that elevates the Hsp90β expression. We proposed that the change in microenvironmental factors including extracellular matrix, metabolites, and inflammatory factors during muscle regeneration and aging might contribute to the elevation of Hsp90β. Nonetheless, how these potential multitude regulatory machineries operate awaits further in‐depth investigation. Our studies, together with others, reveal that there exist multilayer regulatory mechanisms controlling muscle homeostasis and regeneration by different heat‐shock proteins. This obviously provides novel therapeutic targets for muscle‐related diseases.

## 4 EXPERIMENTAL PROCEDURES

See Supporting Information.

## CONFLICT OF INTEREST

The authors declare that they have no conflicts of interest with the contents of this article.

## AUTHOR CONTRIBUTIONS

LLZ, YTZ, and SGY designed and coordinated the study. ZYT, LLZ, LL, QW, and BCL wrote the paper. MYH, SBX, XXC, XCL, and ZHQ performed and analyzed the immunofluorescence assays. MYH, YMG, JFW, SBX, and ZB performed and analyzed the protein–protein interaction and Western blot experiments. MYH and XXC designed and constructed vectors for expression. MYH, SBX, and PL performed and analyzed the RT–qPCR assays. All authors analyzed the results and approved the final version of the manuscript.
