# Supplementary figures and images for: Hsp90β interacts with MDM2 to suppress p53‐dependent senescence during skeletal muscle regeneration
Source: Aging Cell. 2019 Jul 17;18(5):e13003. doi: 10.1111/acel.13003 (PMC6718578; doi:10.1111/acel.13003)

**S1****(a)**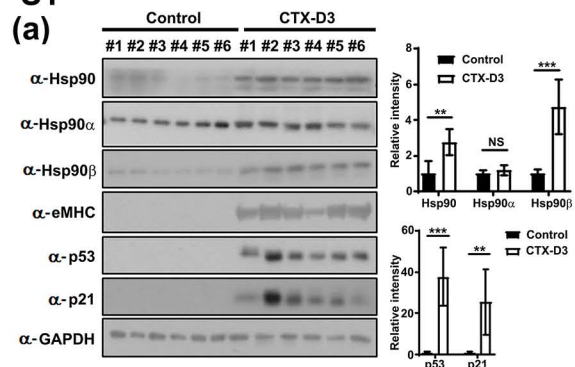**(b)**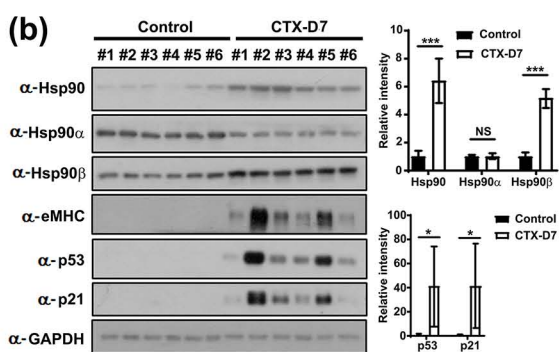**(c)**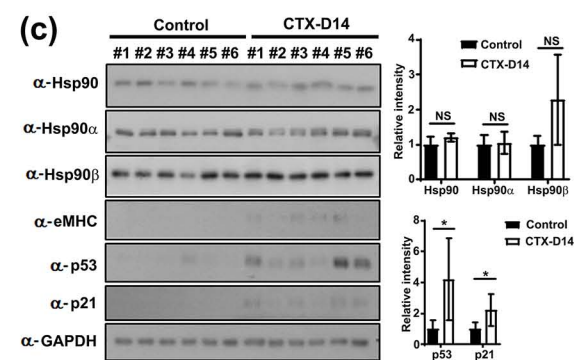**(f)**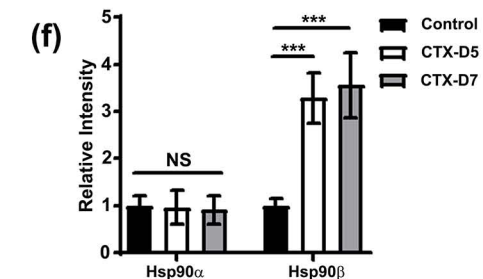**(g)**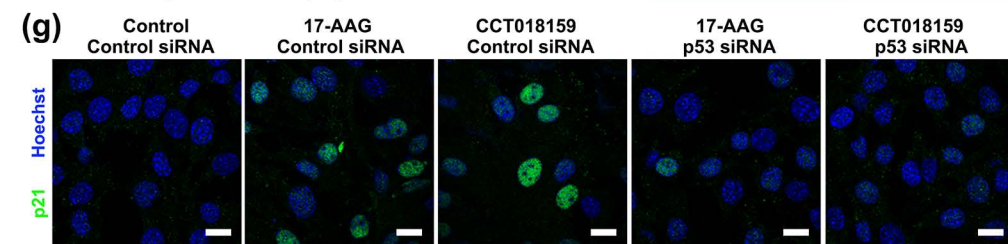**(h)**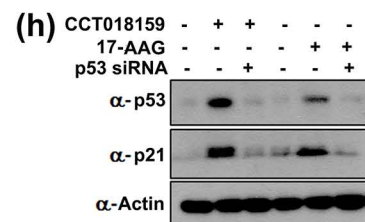**(d)**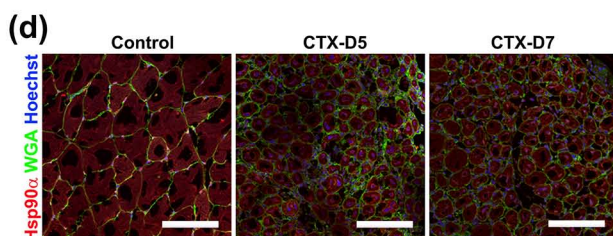**(e)**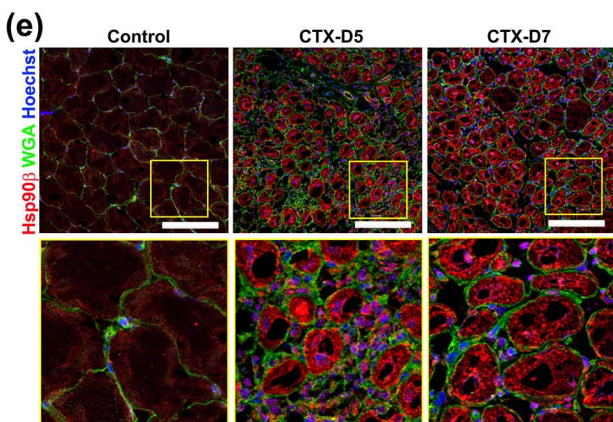

S2

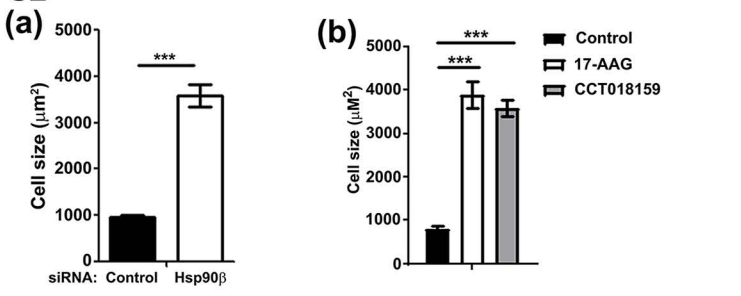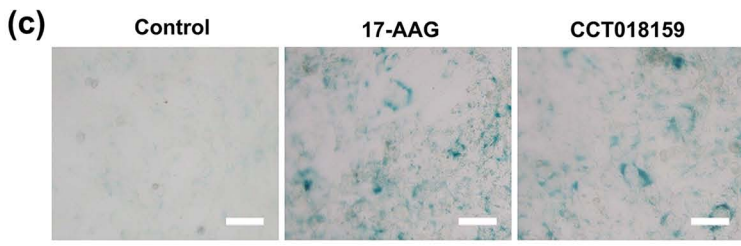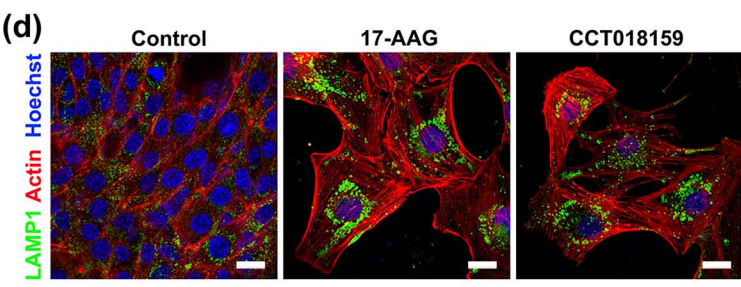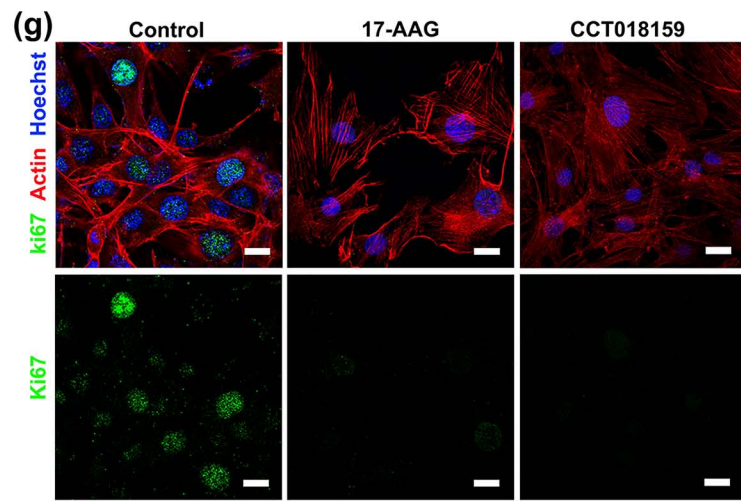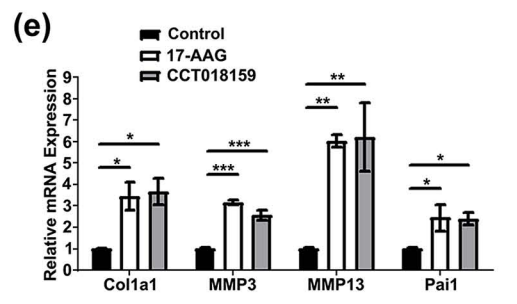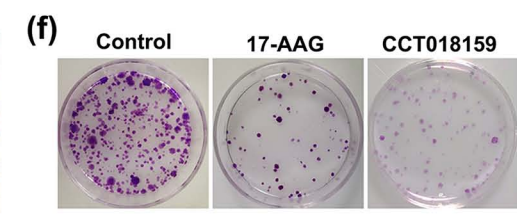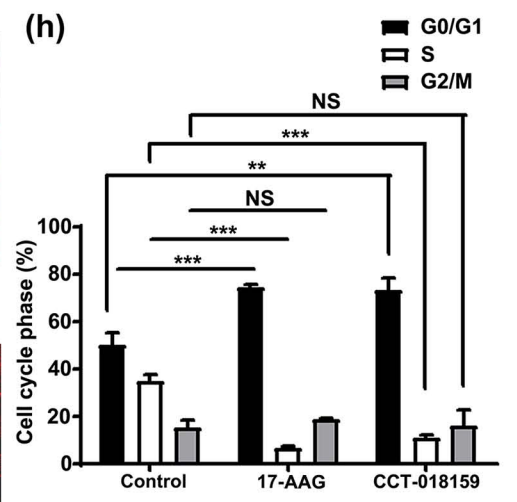

**S3**

**(a)**

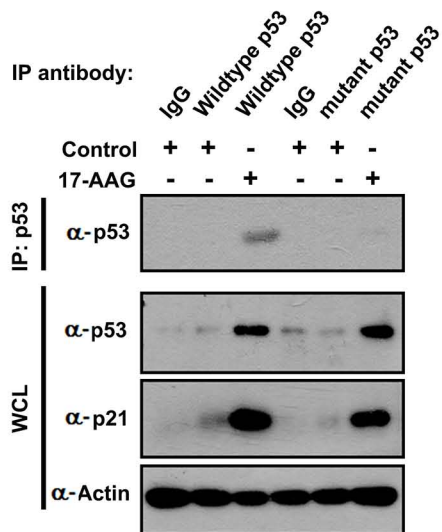

**(b)**

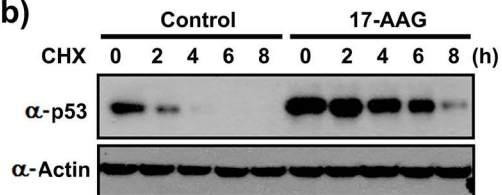

**(c)**

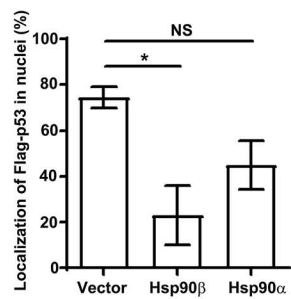

**S4**

**(a)**

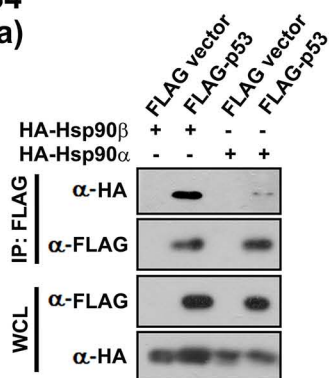

**(b)**

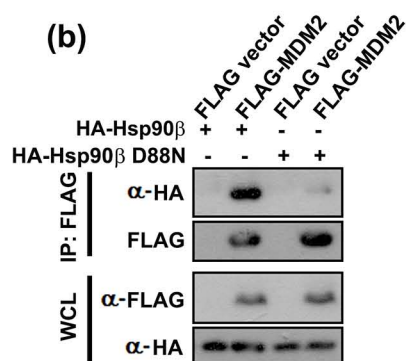

**(c)**

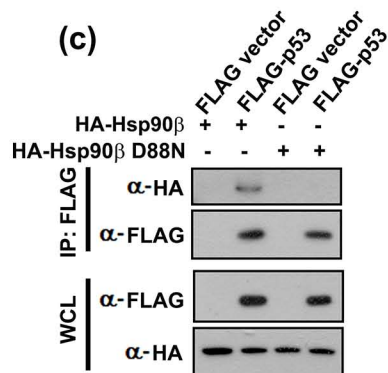

**(d)**

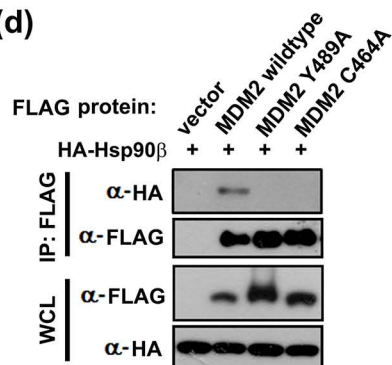

(a)

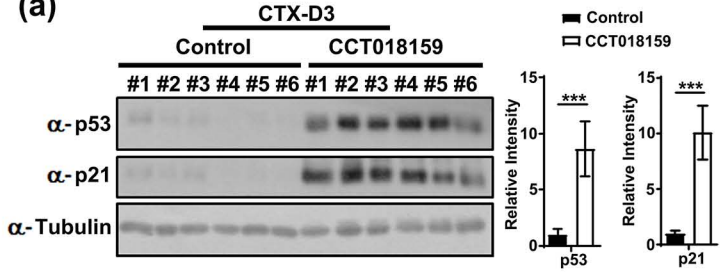

(c)

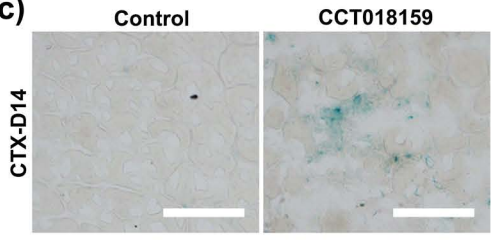

(b)

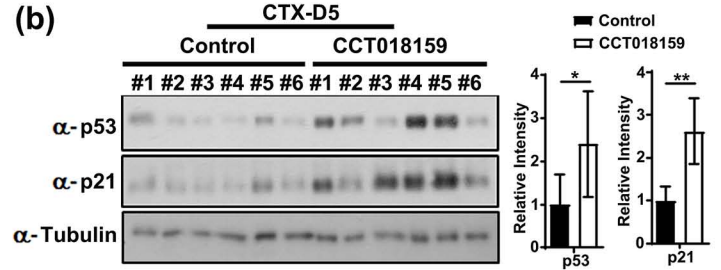

(d)

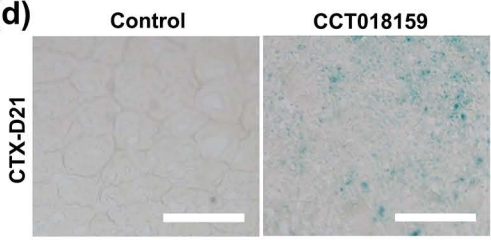

(e)

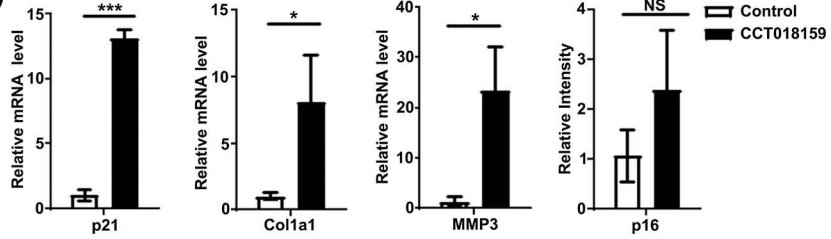

**S6****(a)**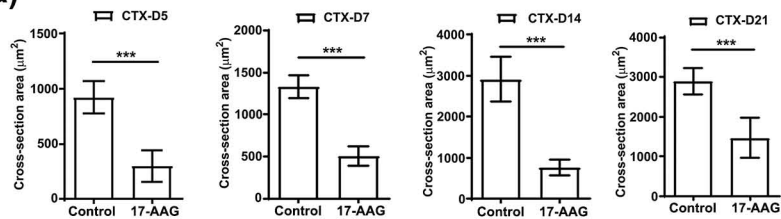**(b)**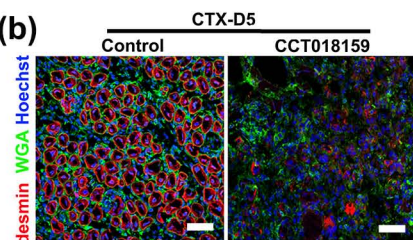**(c)**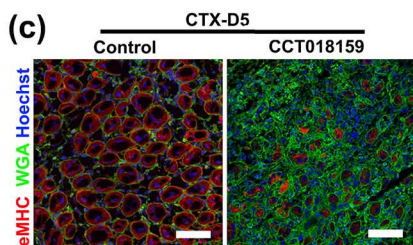**(d)**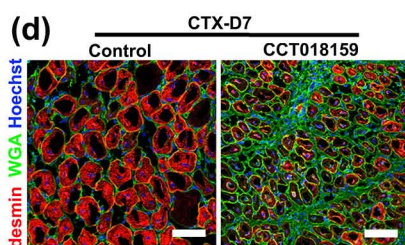**(e)**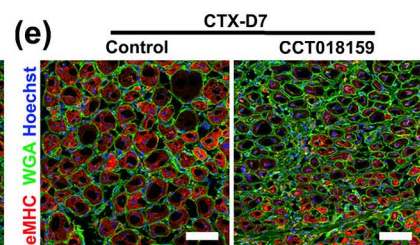**(f)**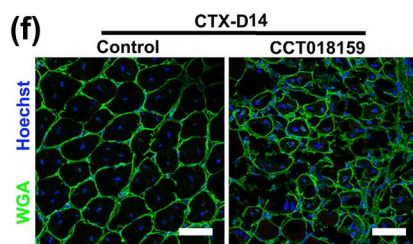**(g)**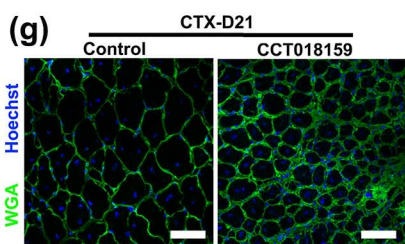**(j)**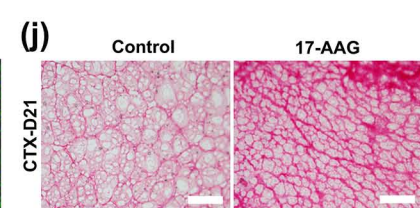**(h)**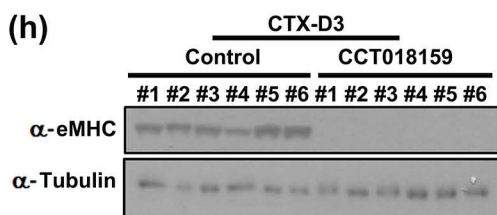**(k)**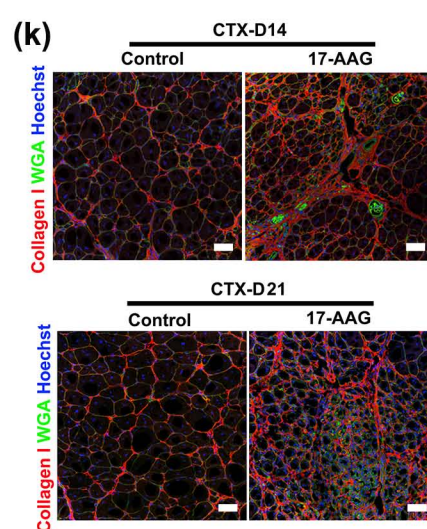**(i)**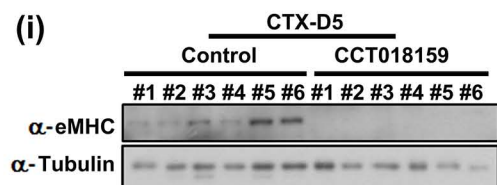



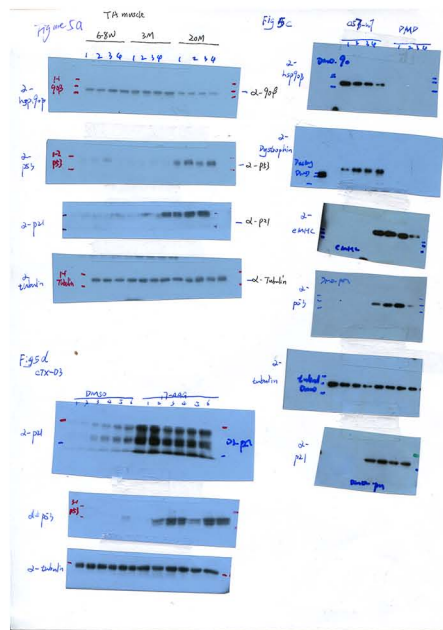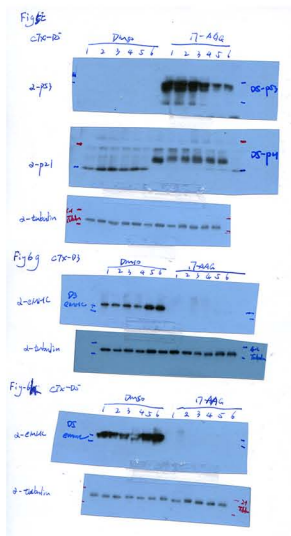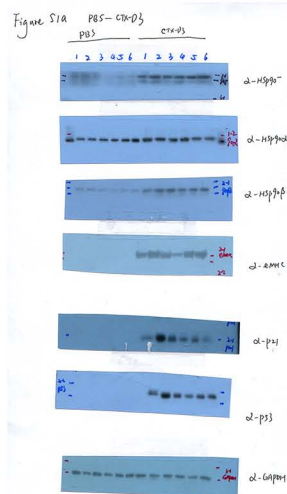

Figure S1b PBS-CTAD7

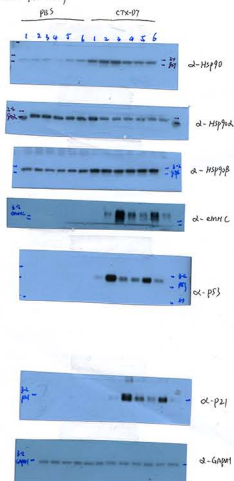

Figure S1c PBS-CTAD4

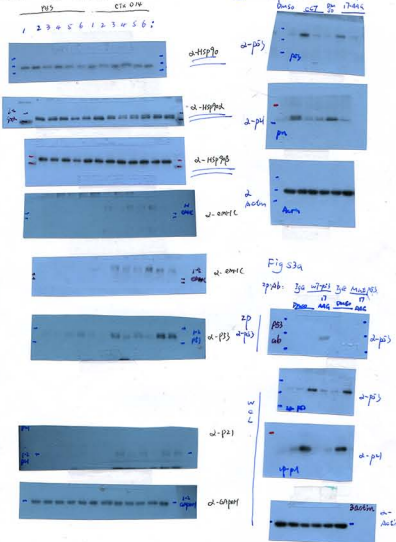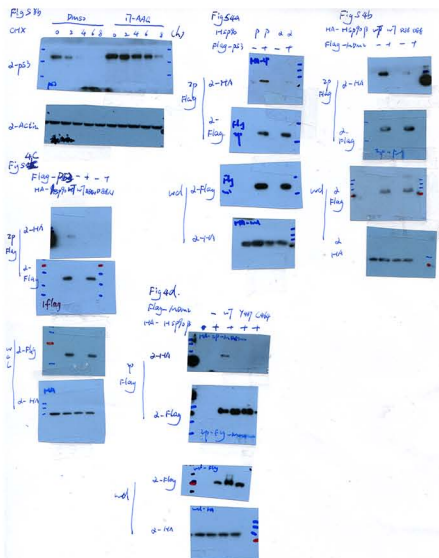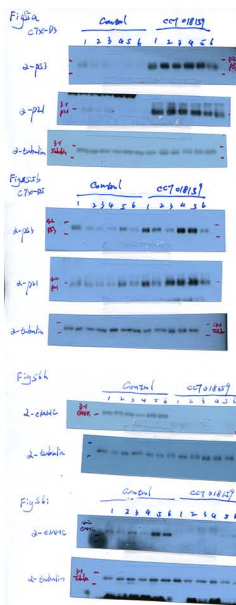

Supplement: Supplementary file 1 [file ACEL-18-e13003-s001.pdf]
