# Supplementary material for: Hsp90β interacts with MDM2 to suppress p53‐dependent senescence during skeletal muscle regeneration
Source: Aging Cell. 2019 Jul 17;18(5):e13003. doi: 10.1111/acel.13003 (PMC6718578; doi:10.1111/acel.13003)
Supplement: Supplementary file 2 [file ACEL-18-e13003-s002.docx]

**Supporting Information**

**Supplemental Experimental Procedures**

**Cell culture and transfection** C2C12 myoblasts were grown in DMEM (high glucose) supplemented with 10% (v/v) fetal bovine serum and Human 293T cells were grown in DMEM medium supplemented with 10% (v/v) fetal bovine serum, 2mM L-glutamine, 100 U/ml penicillin and 100 mg/ml streptomycin (all from Hyclone Laboratories, Logan, UT). Cells in six-well plates were transfected with YoungLipo2000 (NaYang Biotech, Hangzhou) or lipofectamine 2000 (Invitrogen) according to the manufacturer’s protocol.

**Immunoprecipitation studies and Western blot analyses** Control cells or cells transfected with expression plasmids were lysed in HEPES lysis buffer (180 mM NaCl, 1.5 mM MgCl_2_, 5 mM EDTA, 50 mM HEPES, 1% NP40, 10% glycerol, 0.1% sodium orthovanadate, and a mixture of protease inhibitors from Roche Applied Science). Lysates were immunoprecipitated (IP) with anti-FLAG M2 beads (Sigma). The associated proteins were separated on SDS–PAGE and probed with anti-HA or anti-FLAG (for cotransfection experiments).

**Antibody and reagent** Antibodies from Santa Cruz Biotechnology were the following: p21 (sc-53870), Ki 67 (sc-23900), BrdU (sc-32323), p53 (WT) (sc-100), p53 (MUTANT) (sc-99), MDM2 (sc-813). Antibodies from Cell Signaling Technology were the following: p53 (2524S), Desmin (5332S). Antibodies from Developmental Studies Hybridoma Bank were the following: embryonic MHC (F1.652), Lamp 1 (1D4B). Other antibodies were the following: anti-FLAG epitope (Sigma-Aldrich, F7425), anti-HA epitope (Invitrogen, 715500), anti-Tubulin (HuaAn Biotechnology, M1305-2), anti-Actin (HuaAn Biotechnology, M1210-2), anti-Hsp90 (Stressgen, SPA-830), anti-Hsp90β (Stressgen, SPA-844), anti-Hsp90α (Stressgen, SPA-771), anti-Hsp90α (Stressgen, SPA-839), anti-[Collagen](http://www.baidu.com/link?url=K4f7Zh4IHfr2S0tG5R05SVUpq3qUzWtvrKao16zqy-Zt0OfCpReUDizOEcaqJN_CGLvUTigiGBxm-vNVnRToPsr-2TRfDuSjAmnGIsLIHY_) Ⅰ (Abcam, ab21286), anti-p21 (Abcam, ab107099), anti-p27 (BD Biosciences, 610242), anti-p53 (Abcam, ab131442), anti-Dystrophin (Abcam, ab15277).

Chemical reagents used were the following: Wheat Germ Agglutinin, Alexa Fluor™ 488 Conjugate (Thermo Fisher, W11261), Cardiotoxin (Boyao biological, 217504), Phalloidin (Sigma-Aldrich, P1951), Hoechst (Invitrogen, H1399), 17-AAG (Selleck, S1141), CCT 018159 (Santa Cruz, sc-202526), Cycloheximide (Cayman, 14126), MG132 (Santa Cruz, sc-201270), Lactacystin (Santa Cruz, sc-3575), Nutlin-3 (Cayman, 18585)

**Quantitative RT-qPCR** As previously described ([Cong et al., 2018](#_ENREF_10)), total RNA was isolated with RNeasy Kit (Qiagen). Reverse transcription was performed with SuperScript III reverse transcriptase kit (Invitrogen). qPCR reactions using KAPA SYBR FAST qPCR MasterMix kit (kapabiosystems) were performed in triplicate with respective primers as shown in Supporting information Table S1.

**RNA interference** C2C12 myoblasts at 30–40% confluency were transfected with 100 nM of siRNA using Lipofectamine RNAiMAX (Invitrogen) according to the manufacturer’s protocol. Sequences of siRNAs are:

Hsp90β siRNA: 5’-GGAAGAGAAAGGUGAGAAA-3’;

Hsp90α siRNA: 5’-CAGCAAACAUGGAGAGAAU-3’;

p53 siRNA: 5’-GAAUGAGGCCUUAGAGUUA-3’;

p21 siRNA: 5’-GACAAGAGGCCCAGUACUU-3’;

MDM2 siRNA: 5’-GGAACAAGAGACUCUGGUU-3’;

Sequences of control siRNA were 5’-UUCUCCGAACGUGUCACGU-3’.

**RNA-seq Analysis** Total RNA was isolated and used for RNA-seq analysis. cDNA library construction and sequencing were performed by Beijing Genomics Institute using BGISEQ-500 platform. High-quality reads were aligned to the mouse reference genome (GRCm38.p5) using Bowtie2. The expression levels for each of the genes were normalized to fragments per kilobase of exon model per million mapped reads (FPKM) using RNA-seq by Expectation Maximization (RSEM).

**Construction of expression plasmids** Plasmids from Addgene were the following: pcDNA3 FLAG-p53 (#10838) and pcDNA3 MDM2 (#16233). Plasmids from Public Protein *Plasmid* Library (http://www.geneppl.com/linian.php) were the following: pcDNA3-HA-Hsp90β **(**#PPL00024-2a) and pcDNA3-HA-Hsp90α **(**#PPL00023-2a).

For each construct, several clones were chosen and sequenced to the entirety in both directions to confirm their identity. All plasmids were purified using Axygen miniprep kit for use in transfection experiments. *Escherichia coli* strain DH5 was used as host for propagation of the clones.

**Immunofluorescence and direct fluorescence studies** Cells were seeded on coverslips in a six-well plate and transfected with various expression constructs for 24-36 h and then stained for immunofluorescence detection using confocal fluorescence microscopy. FLAG-tagged proteins were detected with monoclonal anti-FLAG followed by Texas Reds or FITCs dye-conjugated goat anti-mouse IgG (Invitrogen). Filamentous actin was detected by rhodamine-phalloidin (Molecular Probes) and microtubule was detected by anti-tubulin (Sigma) followed by Alexa Fluor 488-conjugated goat anti-mouse IgG (Invitrogen). The images were collected with a 63×1.4 NA or 20× objective lens using appropriate laser excitation on Olympus IX81-FV1000 or Zeiss LSM510 Meta laser-scanning confocal microscope. The detector gain was first optimized by sampling various regions of the coverslip and then fixed for each specified channel. Once set, the detector gain value was kept constant throughout the image acquisition process. Images were analyzed with Zeiss LSM Image Examiner Software or FV10-ASW 3.0 Viewer. Cell size were measured by ImageJ of 150 random selected cells from each experimental group.

**Animal Studies** C57BL/6 mice were housed in the animal facility of the Zhejiang University under conventional conditions. All animal experiment protocols were approved by the Review Committee of Zhejiang University School of Medicine and were in compliance with institutional guidelines. For cardiotoxin (CTX) injection, approximately 6-week-old male mice were injected with 50 μl of CTX (10μM) into the tibialis anterior (TA) muscles. To inhibit Hsp90 in vivo, 50 μl of 17-AAG (10μg/ml) or CCT018159 (8μg/ml) were injected into TA at days 1/4, 2, 4, 6, and 10 post-CTX injury. Mice were sacrificed and TA muscles were harvested for Western analyses.

To assess the completion of regeneration and repair, isolated TA muscles were frozen in liquid nitrogen, then frozen in Tissue-Tek CRYO-OCT compound (Fisher Scientiﬁc) and cut in cryostat transverse sections of 8 μm. For immunofluorescence studies, muscle cryosections were fixed with 4% paraformaldehyde, after washing with PBS, antigen retrieval was performed by heating slides in citrate buffer (1.8 mM citric acid and 8.2 mM sodium citrate) at 70℃ for one hour and incubated in blocking buffer (5% goat serum for 1 hour) followed by incubation with the primary antibodies overnight. After washing with PBS, the samples were incubated with Flour Alexa 488- and 546-conjugated secondary antibody (1:200) for one hour at room temperature.

**Cell cycle analysis** Cell suspensions were fixed with 80% ethanol overnight followed by incubating with ribonuclease (RNase) (100mg/ml) and propidium iodide (50 μg/ml). Cellular DNA content was determined by flow cytometry with FACS Calibur (Cytomic FC 500MCL, Beckman Coulter) system and CXP software (Beckman Coulter).

**BrdU incorporation assay** BrdU labeling reagent (1:100; Invitrogen) was added to the culture medium for 4 hours. The myoblasts were then fixed in 4% paraformaldehyde for 30 min followed by treating with 3N HCl for 30 min. After rinsing with PBS, cells were incubated with BrdU antibody (1:500; sc-32323) overnight at 4°C followed by immunofluorescence detection by using confocal fluorescence microscopy. BrdU-positive cells were counted from the entire areas of ten or more culture wells for each experimental group.

**Ubiquitination assay** C2C12 cells were transfected with a hemagglutinin (HA)-ubiquitin construct together with the control vector or FLAG-p53 plasmid or indicated siRNA sequences. Proteasomal inhibitor MG132 at 5 μM was added 6 h prior to harvesting. 48 h after transfection, cells were harvested and lysed in HEPES lysis buffer. The cell lysates were IP with anti-Flag M2 affinity gel. The immunoprecipitates were by western blot analysis using anti-HA or anti-FLAG antibody.

**Protein stability analysis** C2C12 myoblasts transfected with control or Hsp90β siRNA were treated with 100 μg/ml cycloheximide for 0, 2, 4, 6, or 8h. At the indicated time points, the cells were harvested and analyzed by western blot analysis with indicated antibodies. The signal intensity was determined using Image J.

**Colony formation assay** 5×10^2^ C2C12 myoblasts were seeded in triplicate in 6-cm dishes and maintained in DMEM medium supplemented with 10% fetal bovine serum. Growth medium was changed every three days. After 12 days, the resulting colonies were rinsed with PBS, fixed with 4% paraformaldehyde for 10 min, and stained with crystal violet (0.5%).

**Sirius red staining** Sirius red staining was performed following Picro-Sirius Red Stain Kit's instructions (Maokang Biotechnology, MM1004). Sections were stained in picro-sirius red solution for 1 hour, washing in acidified water, dehydrating in 100% ethanol, and then clearing in xylene.

**Supplemental Figure Legends**

**Figure S1. Upregulation of Hsp90β during muscle regeneration.**

(a) Tibialis anterior (TA) muscles of six male mice were injected with CTX and were harvested on day 3 post-injury for Western blot analysis with the indicated antibodies. The expression levels of total Hsp90, Hsp90α, Hsp90β, p53, and p21 were quantified. (b) TA muscles of six male mice were injected with CTX and were harvested on day 7 post-injury for Western blot analysis with the indicated antibodies. The expression levels of total Hsp90, Hsp90α, Hsp90β, p53, and p21 were quantified. (c) TA muscles of six male mice were injected with CTX and were harvested on day 14 post-injury for Western blot analysis with the indicated antibodies. The expression levels of total Hsp90, Hsp90α, Hsp90β, p53, and p21 were quantified. (d) Cryosections of regenerating TA muscles on Day 5- and Day 7-post-CTX (CTX-D5 and CTX-D7) injury were stained with Hoechst for nuclei, Wheat Germ Agglutinin (WGA) for myofiber membrane boundaries, and Hsp90α antibody. Bar: 50μm. (e) Enlarged images of Figure 1b. (f) The expression levels of Hsp90β in myofibers of Figure 1b were quantified. (g) C2C12 myoblasts transfected with control or p53 siRNA were treated with DMSO, 17-AAG, or CCT018159 for 48 hours were subjected to immunofluorescence analysis for p21 expression. Nuclei were visualized by Hoechst staining. Bar: 20μm. (h) C2C12 myoblasts transfected with control or p53 siRNA were treated with DMSO, 17-AAG, or CCT018159 for 48 hours were subjected to Western blot analysis for p53 and p21 expression. (*p < 0.05, **p < 0.01, ***p < 0.001)

**Figure S2. Hsp90 inhibitors induced cellular senescence in myoblasts.**

(a) Quantification of cell sizes of myoblasts from Figure 2a. Cell size were measured by ImageJ of 150 random selected cells from each experimental group. (b) Quantification of cell sizes of myoblasts from Figure 2b. (c) C2C12 myoblasts treated with DMSO, 17-AAG, or CCT018159 for four passages were subjected for SA-βGal staining. Bar: 50μm. (d) C2C12 myoblasts treated with DMSO, 17-AAG, or CCT018159 for three passages were immunostained with Lamp1 antibody. Bar: 20μm. (e) C2C12 myoblasts treated with DMSO, 17-AAG, or CCT018159 for three passages were subjected to qPCR for analyzing Collagen I, MMP3, MMP13, and Pai1 expression. (f) C2C12 myoblasts treated with DMSO, 17-AAG, or CCT018159 for two days were subjected to colony formation assay. (g) C2C12 myoblasts treated with DMSO, 17-AAG, or CCT018159 for two days were stained with Ki67 antibody to analyze proliferation ability. Bar: 20μm (h) C2C12 myoblasts treated with DMSO, 17-AAG, or CCT018159 for two days were subjected to FACS analysis to determine the percentage of cells at different cell phase. (*p < 0.05, **p < 0.01, ***p < 0.001)

**Figure S3. Inhibition of Hsp90 stabilized p53.**

(a) Lysates of C2C12 myoblasts treated with DMSO or 17-AAG for two days were immunoprecipitated with control IgG or antibodies that recognizes wildtype or mutant p53, and then Western blotted with p53 antibody. (b) C2C12 cells were treated with DMSO or 17-AAG for two days followed by treating with cycloheximide (CHX) (100μg/ml). Cell lysates collected at the indicated times were subjected to Western blot analysis. (c) Quantification of nuclear localization of p53 in myoblasts of Figure 3f. (*p < 0.05)

**Figure S4. Identification of Key amino acids mediating the interaction between Hsp90β and MDM2**

(a) FLAG vector or FLAG-p53 were co-transfected with HA-Hsp90α or HA-Hsp90β into C2C12 myoblasts in the indicated combination for 24 hours followed by immunoprecipitation analysis. (b) FLAG vector or FLAG-MDM2 were co-transfected with HA-Hsp90β or HA-Hsp90β D88N mutant into C2C12 myoblasts in the indicated combination for 24 hours followed by immunoprecipitation analysis. (c) FLAG vector or FLAG-p53 were co-transfected with HA-Hsp90β or HA-Hsp90β D88N mutant into C2C12 myoblasts in the indicated combination for 24 hours followed by immunoprecipitation analysis. (d) FLAG, FLAG-MDM2, FLAG-MDM2 Y489A, or FLAG-MDM2 C464A were co-transfected with HA-Hsp90β into C2C12 myoblasts in the indicated combination for 24 hours followed by immunoprecipitation analysis.

**Figure S5. Hsp90 inhibitor CCT018159 induced p53-dependent senescence *in vivo*.** (a) TA muscles with ethanol or CCT018159 injection from six male mice (#1-#6) for each group were harvested on day 3 post-CTX injury for Western blot analysis with the indicated antibodies. The expression levels of p53 and p21 were quantified. (***p < 0.001) (b) TA muscles with ethanol or CCT018159 injection from six male mice (#1-#6) for each group were harvested on day 5 post-CTX injury for Western blot analysis with the indicated antibodies. The expression levels of p53 and p21 were quantified. (*p < 0.05; **p < 0.01) (c) Cryosections of CTX-14 days regenerating TA muscles from ethanol or CCT018159 injected male mice were subjected for SA-βGal staining. Bar: 50μm. (d) Cryosections of CTX-21 days regenerating TA muscles from ethanol or CCT018159 injected male mice were subjected for SA-βGal staining. Bar: 50μm. (e) CTX-14 days regenerating TA muscles from ethanol or CCT018159 injected mice were subjected for RT-qPCR analysis for expression of p21, MMP3, Collagen I, and p16. Data shown are representative of three biological replicates. Statistical analysis was performed with Student’s t test (*p < 0.05, **p < 0.01, ***p < 0.001).

**Figure S6. CCT018159 impaired muscle regeneration.** (a) Quantification of section area (CSA) of regenerating myofibers in control and 17-AAG injected muscles on day 5, day 7, day14, and day21 post-CTX injection. (b) Cryosections of CTX-5 days regenerating TA muscles from control or CCT018159 injected mice were stained with Hoechst for nuclei, WGA for myofiber membrane boundaries, and desmin antibody for newly formed myofibers. Bar: 50μm. (c) Cryosections of CTX-5 days regenerating TA muscles from control or CCT018159 injected mice were stained with Hoechst for nuclei, WGA for myofiber membrane boundaries, and embryonic myosin heavy chain (eMHC) antibody for newly formed myofibers. Bar: 50μm. (d) Cryosections of CTX-7 days regenerating TA muscles from control or CCT018159 injected mice were stained with Hoechst for nuclei, WGA for myofiber membrane boundaries, and desmin antibody for newly formed myofibers. Bar: 50μm. (e) Cryosections of CTX-7 days regenerating TA muscles from control or CCT018159 injected mice were stained with Hoechst for nuclei, WGA for myofiber membrane boundaries, and eMHC antibody for newly formed myofibers. Bar: 50μm. (f) Cryosections of CTX-14 days regenerating TA muscles from control or CCT018159 injected mice were stained with Hoechst for nuclei and WGA for myofiber membrane boundaries. Bar: 100μm. (g) Cryosections of CTX-21 days regenerating TA muscles from control or CCT018159 injected mice were stained with Hoechst for nuclei and WGA for myofiber membrane boundaries. Bar: 100μm. (h) TA muscles with ethanol or CCT018159 injection from six male mice (#1-#6) for each group were harvested on day 3 post-CTX injury for Western blot analysis with eMHC. (i) TA muscles with ethanol or CCT018159 injection from six male mice (#1-#6) for each group were harvested on day 5 post-CTX injury for Western blot analysis with eMHC. (j) Sirius Red staining of cryosections of CTX-21 days regenerating TA muscles from control or 17-AAG injected mice. Bar: 100μm. (k) Collagen I staining of cryosections of CTX-14 days and CTX-21 days regenerating TA muscles from control or 17-AAG-injected mice. Bar: 50μm. (***p < 0.001)

**Figure S7. Original gel films of Figure 1a, 1e, 1i, 1j, 1l, 3a, 3c, 3d, 3e, 3g, 4a, 4b, 4d, 4e, 4f.**

**Figure S8. Original gel films of Figure 4h, 4i, 4j, 4k, 5a, 5c, 5d, 5e, 6g, 6h, S1a.**

**Figure S9. Original gel films of Figure S1b, S1c, S1h, S3a, S3b, S4a, S4b, S4c, S4d, S5a, S5b, S6h, S6i.**

**Table S1: Primers used in Quantitative RT-qPCR study**

| **Gene** | **Forward Primer** | **Reverse Primer** | **Accession No.** |
| --- | --- | --- | --- |
| p21 | GTGGGTCTGACTCCAGCCC | CCTTCTCGTGAGACGCTTAC | NM_007669.5 |
| p27 | TCAAACGTGAGAGTGTCTAACG | CCGGGCCGAAGAGATTTCTG | NM_009875.4 |
| Col1a1 | CCCTGGTCCCTCTGGAAATG | AGCTGGACACGGAGCTTTTA | NM_007742.4 |
| MMP3 | CCTGATGTTGGTGGCTTCA | TCCTGTAGGTGATGTGGGATTTC | NM_010809.2 |
| MMP13 | ACTTCTACCCATTTGATGGACCTT | AAGCTCATGGGCAGCAACA | NM_008607.2 |
| Pai1 | TCAGAGCAACAAGTTCAACTACACTGAG | CCCACTGTCAAGGCTCCATCACTTGCCCCA | NM_008871.2 |
| IL-8 | GACGAGACCAGGAGAAACAGGG | AACGGAGAAAGAAGACAGACTGCT | NM_011339.2 |
| IL-6 | ATGCTCCCTGAATGATCACC | TCACAGATGGCGTTGACAAG | NM_031168.2 |
| p53 | GGCGTAAACGCTTCGAGATG | CTTCAGGTAGCTGGAGTGAGC | NM_011640.3 |
| p16 | CGTACCCCGATTCAGGTGAT | TTGAGCAGAAGAGCTGCTACGT | NM_009877.2 |
